# The use of Qualitative Comparative Analysis (QCA) in child well-being research: a scoping review of research on child well-being research and interventions

**DOI:** 10.1186/s12889-025-23821-x

**Published:** 2025-09-25

**Authors:** Aye Thiri Kyaw, Meghna Ranganathan, Cathy Zimmerman, Isabelle Pearson, Emily Warren, Benjamin Hanckel

**Affiliations:** 1 Department of Global Health and Development, Gender Violence and Health Centre, LSHTM, London, UK; 2 Department of Health Services Research and Policy, LSHTM, London, UK; 3 Institute for Culture and Society, Western Sydney University, Sydney, Australia

**Keywords:** Child well-being, Children, Qualitative Comparative Analysis (QCA)

## Abstract

**Background:**

Qualitative Comparative Analysis (QCA) is a method for examining configurational causality by identifying pathways that lead to an outcome of interest. There is a growing body of literature that uses QCA to measure child well-being due to its ability to generate evidence of causality for complex social phenomena. This scoping review examines how QCA studies are being employed to investigate child well-being and assesses the potential of QCA as a method to produce intervention-focused evidence and to contribute to future methodological development to address the complexity of child well-being.

**Method:**

We systematically searched Embase, PsyINFO, MEDLINE, Social Policy and Practice, Global Health, Econlit, Scopus and Web of Science for peer-reviewed studies that had used QCA methods in child well-being studies. We searched studies published in English up until 2023. Systematic reviews and meta-analyses using QCA were excluded due to insufficient methodological detail for inclusion in our analysis. We followed the PRISMA-ScR flowchart and guidelines for study screening to ensure a systematic selection process. Data extraction was undertaken to capture information of most relevance to QCA best practice. Data were analysed using a basic qualitative content analysis approach.

**Results:**

The search identified 626 papers, of which 28 met our inclusion criteria. Dimensions of well-being included: psychological/mental health (*n* = 9); physical health (*n* = 2); language development under education (*n* = 1); socio-emotional health (*n* = 7); physical and psychological/mental health (*n* = 3), psychological/mental health and education (*n* = 1); and multi-dimensional health (*n* = 3). Two studies stated explicitly that they used well-being concepts—subjective well-being and psychological well-being. Most studies (*n* = 23) were predominantly in high income countries (HIC). Commonly reported strengths of QCA were the capacity to a) describe various pathways or combinations of pathways to the same outcome (equifinality); and b) examine conjunctural causation (combination of absent/present conditions), known as ‘causal complexity’. Weaknesses related to a) generalisability of the data; and b) the number of causal conditions that can be included in the analysis. Our findings suggest that QCA can be effectively used alongside traditional analyses to provide a more nuanced understanding.

**Conclusion:**

QCA is a promising method with potential to address complexity when assessing the different dimensions of child well-being. More comprehensive guidelines are now available that offer good practices to enhance the quality of the QCA research. To build greater confidence using this method, scholars are recommended to adhere to these good practices to establish the highest levels of transparency of the analysis.

**Supplementary Information:**

The online version contains supplementary material available at 10.1186/s12889-025-23821-x.

## Introduction

Qualitative Comparative Analysis (QCA) is a method that examines the configurational conditions that lead to an outcome of interest [1]. There is a growing body of literature using QCA to measure child well-being and evaluate interventions [2–6]. Scholars of child well-being consistently note the need for understanding and making sense of the multiple aspects of well-being as an outcome across contexts [1, 6, 7]. However, there is limited information on how this emergent method is being used across this field. The primary aim of this scoping review is thus to examine how QCA studies are being employed to investigate child well-being in low- and middle- income countries (LMIC) and high-income countries (HIC).

### Child well-being

Well-being is a broad concept that has been operationalised in different ways across child well-being studies [8, 9]. In the child well-being literature, one common way to conceptualise child well-being is to classify it into objective and subjective well-being [10]. Objective well-being relates to external conditions to the individual, such as income, literacy and life expectancy that can be measured using a variety of validated indicators [11]. In contrast, subjective well-being refers to individuals’ perceptions and experiences [11, 12]. The subjective aspect of well-being is classified by its two main historical roots: hedonic well-being and eudaimonic well-being. Hedonic well-being emphasises happiness, positive and negative emotions, and life satisfaction. In contrast, eudaimonic well-being relates to experiences of personal functioning and the pursuit of meaningful goals and self-actualisation [13].

The current literature on children’s well-being acknowledges that children’s needs are multidimensional [14]. For instance, in their systematic review of published child well-being studies between 1991 and 1999, Pollard and Lee (2003) identified five distinct dimensions of well-being: physical, social, psychological, cognitive and economic as critical components contributing to a child’s overall well-being and development [8]. Indicators of child well-being for the physical dimension include physical activities, wellness knowledge such as nutrition awareness and personal hygiene practices. Indicators for the psychological dimension include emotions, mental health including depression, and/or illness. Indicators for the cognitive dimension include intellectual and school-related activities. Indicators for the social dimension include family relationships, emotional support, socially desirable behaviours, and communication skills. Indicators for the economic and financial dimension include financial security [8].

A more recent review of measurement tools for child well-being studies (2000–2019) elaborated on this prior conceptualisation, proposing well-being dimensions that include physical health and safety; behaviours and risks/safety, housing, environment, and neighbourhood; social relationships, psychological health and socio-emotional well-being [9]. This conceptualisation also connects to the World Health Organization (WHO) definition that indicates that well-being is influenced by social, economic, and environmental determinants of health. In this way, wellbeing is a multi-dimensional concept. This paper acknowledges child well-being as a multidimensional concept encompassing all the elements presented. On 12 June 2023**,** scoping review protocol (see Additional file 1) was registered at Open Science Framework (OSF).

### Qualitative Comparative Analysis

An approach increasingly used to examine child well-being is QCA [2, 6, 15, 16]. Designed by Ragin in the late 1980s, it is a case-based methodological approach that explores relationships between ‘conditions’ (similar to variables) and ‘outcomes’ across cases [17]. While QCA has been in existence for the last several decades, its adoption in research has been relatively limited until recent years. A range of factors, such as its perceived methodological complexity, limited software availability in earlier years, and a lack of familiarity among researchers may have contributed to its slower uptake [18, 19]. Although QCA contributions were initially found in the fields of political science, sociology, and economic and management studies, it has become increasingly common in public health [20–22]. QCA approaches often are exploratory, but can also be used to evaluate theory, using for example, realist approaches [23]. In QCA, conditions are the factors or variables hypothesized to influence a specific outcome. QCA combines qualitative in-depth knowledge of cases (which may be individuals, classrooms, schools, countries, etc.) with quantitative approaches to identify empirical patterns across cases. QCA was originally developed for small- and medium-N research; however, it has been effectively extended to Large- N research [24, 25]. Approaches to cases can be either case-oriented or condition-oriented. There has been increased use of large-N QCA with a condition-oriented approach to understand the conditions and patterns between the cases without using in-depth case information [23, 26].

QCA offers several strengths for examining complex systems and casual complexity. This includes QCA’s ability to assess equifinality, or the various pathways to the same outcome. Second, it accounts for conjunctural causation, which examines the combination of absent/present conditions in relation to each other and could be key to the same outcome of interest [1, 27]. For instance, if well-being is the outcome of interest, undertaking a QCA would enable a researcher to examine the various combinations of conditions (or ‘causal recipes’) that may lead to well-being across cases (individual, school, countries etc.) in which there might be more than one casual recipe that results in well-being. This provides us with a tool to surface the ways different conditions produce similar outcomes across cases of interest.

Using set-theory, QCA aims to identify the conditions that are ‘necessary’ or ‘sufficient’ for the outcome of interest to emerge [1]. For a condition to be necessary, it must be always present when the outcome is present. In contrast, a condition is sufficient when the outcome of interest is present, yet the outcome could still result from other conditions. In most QCA studies, as Legewie [27] argues, conditions or combinations of conditions are ‘quasi-necessary’ or ‘quasi-sufficient’ for the outcome. Two further distinctive features of QCA include the assessment of *consistency* and *coverage*. Consistency assesses the degree to which cases with the same combinations of conditions produce the same outcome. Coverage assess the degree to which a combination of conditions is responsible for the occurrence of the outcome [1].

Potential causal conditions for inclusion in QCA models are selected by drawing on knowledge of the cases and the research literature. The selection of conditions should be iterative and be driven by relevant theory, empirical evidence, and the researchers’ judgement [26]. Good practice suggests keeping the number of conditions at a moderate level, as having many conditions can make the interpretation of results difficult [28].

After relevant conditions are selected for exploration, they need to be calibrated. During calibration, a researcher with deep knowledge of all the cases assigns what are known as ‘membership scores’ to the cases, showing whether each case is fully in the membership or out of the membership. There are two main variants of QCA: crisp-set QCA (csQCA) and fuzzy-set QCA (fsQCA). Multi-value QCA (mvQCA) also exists but is less frequently used. CsQCA is used in conventional sets where cases are either in or out of membership sets. FsQCA allows researchers to calibrate partial membership in sets (set of causal conditions and outcome) using values in the interval between 0 (non-membership) and 1 (full membership) [1].

In QCA, one of the key tools for examining causal complexity is called the ‘truth table’, which identifies explicit connections between combinations of causal conditions and the outcome [1]. The truth table lists all the logically possible conditions designated by the researcher, during which, using Boolean algebra, a researcher specifies if a condition is absent/present in each case. The truth table generates three types of solutions: complex, parsimonious, and intermediate. These solutions reflect the combinations of conditions leading to the outcome but differ in the level of detail describing these combinations [29].

Scoping reviews are a type of knowledge synthesis that responds to an exploratory research question by systematically searching, selecting, and synthesising existing knowledge in order to map the key concepts, types of evidence, and research gaps related to a defined field or area [30, 31]. While QCA has been used to assess child well-being, to our knowledge, no review on its usefulness has been published. Thus, we undertook a scoping review on the use of QCA in child-well-being studies and an assessment of how the method has been used to measure the complex and heterogeneous nature of child well-being. This review was guided by the research question: *How has QCA been used in child well-being research and for interventions?* In addition, the review aims to identify the reported strengths and limitations of QCA approaches; and examine the gaps and implications for future child well-being research and interventions.

## Method

This scoping review drew on methods from the Arksey and O’Malley framework [30] and followed the PRISMA-ScR (Preferred Reporting Items for Systematic reviews and Meta-Analyses extension for Scoping Reviews) developed by Tricco et al. [32].

### Search strategy and selection criteria

We searched for studies published in any language with an English abstract, published between 2000 to 2023. While the literature using QCA in LMICs is expanding, it remains relatively limited in the child well-being thematic area. To address this, we included studies from high-income countries (HICs) to strengthen the evidence base. Studies were included if they had participants under 18 years old and employed variants of QCA (csQCA, fsQCA or mvQCA) that reported one or more dimensions of child well-being. For well-being outcomes, we included studies that assessed one or multiple dimensions of child-wellbeing as an outcome, that had as their unit of analysis individual well-being or interventions with well-being as an outcome. Systematic reviews/meta-analyses [20–22, 33–36]that apply QCA were excluded as it does not provide the granular methodological details necessary for our analysis (see Additional file 4). We intentionally focused on empirical studies as our aim was to examine the methodological use, implementation processes, and context-specific insights that are typically detailed in empirical QCA studies. Grey literature was also excluded.

Our search terms included a combination of the following: **“**Qualitative Comparative Analysis”**, “**Child”, “Adolescent” and** “**Well-being” (see Additional file 2). ‘Child’ or ‘children’ are broadly defined in this study as individuals under 18 years of age, in accordance with UNICEF’s definition. Informed by available systematic reviews on child well-being [8, 9], well-being is defined as multi-dimensional concept, where these dimensions are generally concerned with the areas: 1) Health/physical health and safety; 2) education; 3) economic and material well-being; 4) behaviours and risks/safety; 5) housing, environment, and neighbourhood; 6) social relationships; 7) psychological health and socio-emotional well-being as well as subjective well-being, which usually covered self-defined health, well-being at school, and personal well-being; objective well-being and capability.

We used the following eight databases: Embase, PsyINFO, MEDLINE, Social Policy and Practice, Global Health, Econlit, Scopus and Web of Science. The reference lists of ten randomly selected relevant manuscripts from the broader sample were manually searched to identify any further relevant studies. A ‘snowball’ technique was also adopted in which citations within articles were searched if they appeared relevant to the review.

### Screening

We used Rayyan for the screening process to streamline study selection and ensure consistency throughout the review [37]. A two-staged screening method was used to determine the relevance of studies obtained through the search. For the first level of screening, we only assessed the title and abstract. When the abstract of the study could not be found, the study was included to the full text screen stage. The titles and abstracts of all studies were screened by ATK (100%) and IP (10%). Full texts of the 28 remaining studies were screened by ATK for inclusion, with five papers double screened by ATK and IP. Papers relating to "quantitative coronary angioplasty" and "qualitative content analysis" (both of which also abbreviated as "QCA") were among the excluded studies. Other papers reported methodological issues and background information but did not include empirical studies. Finally, papers that used the term "qualitative comparative analysis" to refer to qualitative studies that compared various sub-populations or cases within the study but did not include formal QCA methods were also excluded.

### Data extraction and analysis

The following data were extracted: study information, rationale for using QCA and information on QCA, including strengths and limitations. Following the first round of data extraction procedures [20], our team (ATK, BH, MR, CZ, and IP) went back and also extracted data about whether the calibration of set-membership scores was discussed and justified by authors, their familiarity with the cases, whether they report the truth table and data matrix, and whether the reported solution formula and consistency/coverage values were included in reporting. The first author manually extracted data from 28 full texts following QCA reporting guidelines. To ensure accuracy and consistency, BH, MR, and CZ independently reviewed 100% of the data extractions, while IP cross-checked 10%. Two papers relating to the same research question and data set were combined, such that analysis was by study (n = 28), not by paper. Data were synthesized by using thematic analysis, guided by the framework developed by Hanckel et al. 2021 [20], while also reflecting on the JBI guidance on presenting the findings [38].

### Assessing indicators of reporting quality

There are no reporting guidelines on the best ways to report QCA. Existing guidance suggest best practices such as making the raw or calibrated dataset accessible, publishing the truth table, reporting all thresholds for consistency and coverage measures, and providing the solution formulas along with the parameters of fit [23, 24, 28]. It also offers clear and explicit calibration rules and/or justification for assigning set membership scores to the cases, drawing both on theoretical and empirical information [28]. We applied the following reporting criteria used by Hanckel et al. in their systematic review of QCA on public health interventions to guide the assessment of best practice in QCA reporting [20].

i) Inclusion of calibration of set membership scores being discussed in detail and justified;
ii) Shows the familiarity of the cases;
iii) Reports an explicit and detailed justification for selection of cases;
iv) Reports the truth table and raw matrices;
v) Contains analysis with the solution formula; and
vi) Reports the consistency and coverage measures;

## Results

**Fig. 1:**
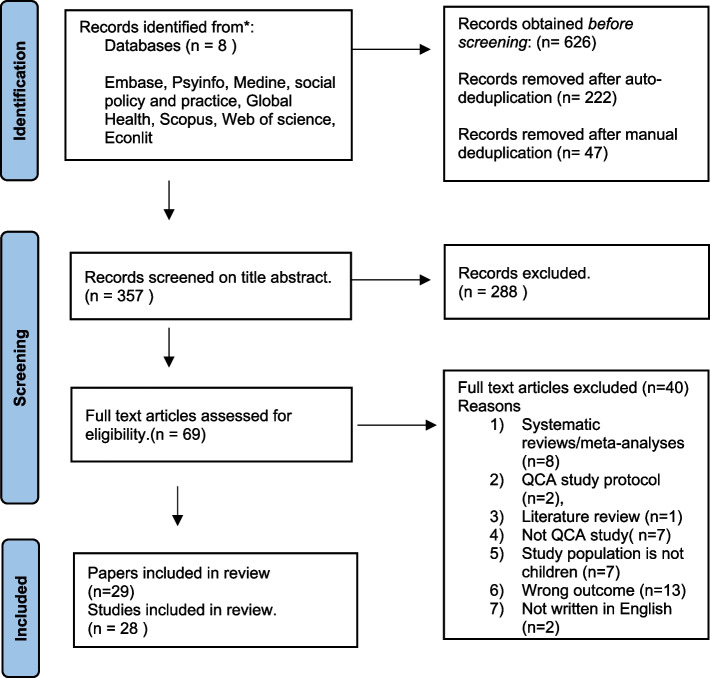
Prisma flow chart of screening and selection process

The search returned 626 studies. After de-duplication, 357 studies remained, and abstracts were screened. The abstract screening identified 69 peer-reviewed articles for full text screening. At the full-text stage, 40 were excluded and 29 remained for inclusion (see Fig. 1 Prisma flow chart of screening and selection process). Two papers provided results of the same study [40, 41], so both are included, but are hereafter counted as one study, leaving 28 separate studies. The results are reported in accordance with the checklist of preferred reporting items for systematic reviews and meta-analyses extension for Scoping Reviews (PRISMA-ScR) (see additional file 3).

### Study characteristics of QCA research in child well-being

The earliest study was published in 2013 [40], eight studies were published in 2015–2019 and 17 studies were published after 2020, suggesting the growth in the use of QCA. A summary of the study characteristics drawn from the 28 included studies is presented in Table 1.

**Table 1:** Studies included in the review: summary of data extracted

Publication details		Study data extracted								
**Lead author (Date)**	**Aim of the study**	**Well-being dimension**	**Rationale of using QCA**	**Sole QCA or with additional analysis**	**Type of data and study used for analysis -source**	**Population & country settings**	**Sampling method**	**Number of cases (potential cases) and level of the cases**	**Number of conditions**	**outcome (phenomena explained)**
Schoeps 2020 [45]	Analyse the combined effect of parenting styles and psychopathological symptoms to the prediction of personal, social, and school adaptation in school children, accounting for potential sex and age differences	Psychological/mental health and socio-emotional health	"To establish different combinations between the conditions rather than assuming highly particularized and in‐depth descriptions as do the hierarchical linear regressions"	FsQCA with hierarchical linear regression	Primary quantitative data (Cross-sectional study)	Children from 5 and 6th Grade of Primary Schools. (Age—10,11,12), Valencia region, Spain	Purposive sampling	367 (Individual level)	7	Child adaptation (Personal, School and Social maladjustment)
Gao 2020 [44]	Investigate conditions that contributed to high scores on smartphone use disorder in adolescents	Psychological/mental health	"The adoption of fsQCA is suitable in the current study because different psychosocial factors in adolescents have, instead of an added sum of individual net effects, complex integrated effects on developmental outcomes"	FsQCA with regression analysis	Primary quantitative data (Cross-sectional study)	Students from grade 5–11 from six schools, China	Not reported	1766 out of 1769 (Individual level)	6	Smart phone use disorder
Herrera 2022 [51]	Understand the predictors of life satisfaction among adolescents in Ecuador during confinement	Subjective well-being	Not reported	FsQCA with Structural Equation Modelling (SEM)	Primary quantitative data (Cross-sectional study)	Adolescents (age 12–18), Ecuador	Not reported	902 out of 1355 (Individual level)	7	Life satisfaction
De la Barrera 2019 [41]	Analyse the combined contribution of emotional awareness, moods, and personality in the somatic complaints of healthy children and adults using QCA	Physical and Psychological/mental health	“A fuzzy-set qualitative comparative analysis (fsQCA) allows for conjunctions of all logically possible combinations of conditions"	Sole FsQCA	Primary quantitative data (Cross-sectional study)	Children from 12 public, subsidised and private schools (Age: 8–14), Valencia region, Spain	Convenience sampling	1476 (Individual level)	4	Somatic complaints
Coello 2022 [5]	Understand predictors of emotional distress among adolescents in Ecuador during confinement	Psychological/mental health	Not reported	FsQCA with SEM	Primary quantitative data (Cross-sectional study)	Adolescents from Ecuador	Convenience sampling	586 adolescents out of 902 (Individual level)	7	Emotional distress
Gulbas 2019 [57]	Explore the cultural and developmental appropriateness of Inter-Personal Psychological Theory of Suicide within a sample of Latina adolescents who attempted suicide	Psychological/mental health	"To analyse how different configurations of codes relate to the presence or absence of a phenomenon"	Sole QCA	Primary qualitative data (Cross-sectional study)	Latina adolescents, New York city, United States	Not reported	60 (30 with attempted suicide and 30 without it), (Individual level)	3	Evaluating Interpersonal Psychological theory of Suicide
Liu 2022 [52]	Examine how marital conflict influences juvenile delinquency through its spill over influences on mental health problems and delinquent peer association among Chinese adolescents	Psychological/mental health	"The current study supplements SEM analysis with fsQCA, which can provide combinations of variables to improve outcomes and allow for a casual analysis of how marital conflict, youth mental health problems, and delinquent peer association jointly contribute to delinquency"	FsQCA with SEM	Primary qualitative and quantitative data (cross-sectional study)	Secondary school students, Metropolitan area in China	Probabilistic sampling	3047 (Individual level)	3	Juvenile Delinquency
Villanueva 2017 [42]	Examine trait emotional intelligence and feelings as predictors of biological and perceived stress in a sample of healthy adolescents	Psychological/mental health and physical health	"Qualitative comparative analysis (QCA), allows for an in-depth analysis of how causal conditions contribute to an outcome. QCA also considers equifinality, that is, different ways to arrive at a particular result"	FsQCA with hierarchical linear regression	Primary quantitative data (cross-sectional study)	Pupils from two public and two private schools (Age 12–14), Valencian region, Spain	Convenience sampling	170 (Individual level)	6	Biological or perceived stress
Short 2020 [60]	Investigate the combinations of factors (paths) that result in good and poor language outcomes for a group of 5-year-old children of mothers experiencing adversity	Language development	“We chose QCA as our method for this study because it has the capacity to deter- mine whether there are complex paths to the language outcomes in children of mothers who received home visiting intervention.”	Sole Crsipset QCA	Secondary quantitative data (cross-sectional study)	41 with or without optimal intervention, New South Wales, Australia	Not reported	41 (Individual level)	8	Good or poor language outcomes
Mei 2022 [53]	Compare the performance of the regression models and fuzzy set qualitative comparative analysis (fsQCA) models in analysing the possible effects of socio demographic variables and lifestyle behaviours on depressive symptoms in adolescents	Psychological/mental health	"Adolescent health related risk behaviours in life often do not exist alone, but present gathered or phenomenon. Therefore, fsQCA was used in this study to explore the effect of the combination of different behaviours patterns on depression symptoms"	FsQCA with hierarchical linear regression	Primary quantitative data (cross-sectional study)	800 adolescents from 8 junior high schools and 3 senior high schools, Jillin Province, China	Random cluster sampling	800 (Individual level)	7	Depressive symptoms
Jimenez-Rodriguez 2022 [49]	Deepen understanding of the context of vulnerability which is associated with drug-taking behaviour and addiction	Psychological/mental health	"Fuzzy set qualitative comparative analysis (fsQCA) was used to explore a variety of different paths that combine multiple predictors in different ways"	FsQCA with SEM	Primary quantitative data (Longitudinal study)	Adolescents from the first and fourth year of Compulsory Secondary Education (ESO) in different educational centres (age-12–17), valencian region, Spain	Not reported	815 (Individual level)	6	Psychological adjustment
Villanueva 2022 [43]	Analyse the combined contribution of trait emotional intelligence, self-esteem, and perceived stress to various indicators of preadolescents’ well-being	Physical and Psychological/mental health	Not reported	FsQCA with hierarchical linear regression	Primary quantitative data (Longitudinal study)	Pupils from public and private high school. (Age 12–16), Valencian region, Spain	Convenience sampling	381 (Individual level)	7	Subjective well-being is assessed by life satisfaction and somatic complaints
Wilhelmsen 2019	Capture the motivational mechanisms involved in inclusion in physical education	Physical health	"QCA is well suited for grasping complex and asymmetric relations compared with traditional statistical inference, which has been commonly used in the literature"	Sole FsQCA	Primary quantitative data (cross-sectional study)	Children with disabilities (Age 7–16), Norwegian elementary schools (Grade 2–10)	Convenience sampling	64 (Individual level)	7	Social and Pedagogical Inclusion in Physical Education
Kien 2018 [56]	Assess pathways to successfully achieving high SCE through a physical activity intervention for primary school children	Socio-emotional health	"We chose QCA as the method of analysis, as we investigated a small number of cases and aimed to answer a question related to the combinations of conditions and not related to the identification of the independent influence of a variable"	Sole FsQCA	Primary qualitative and quantitative data (cross-sectional study)	24 classes participating in health promotion intervention (out of 27 classes), Lower Austria	Not reported	24 (class level)	5	Emotional and social experiences of children
De la Barrera 2019 [41]	Examine the combined contribution of the socio-emotional factors of emotional competence and self-esteem, and the personal factors of sex, age, and number of siblings, in the prediction of psychological adjustment and subjective well-being in adolescence	Socio-emotional health	Not reported	FSQCA with descriptive statistics	Primary quantitative data (cross-sectional study)	Adolescents from 6 public and private high school, autonomous communities of Madrid and Valencia (Spain)	Convenience sampling	840 (Individual level)	7	Emotional and behavioural adjustment; satisfaction with life in adolescent
Valero-Moreno 2020	Studies the psychological characteristics of a group of paediatric patients diagnosed with PCD in comparison with their healthy peers	Psychological well-being	Not reported	Sole FsQCA	Primary quantitative data (cross-sectional study)	Pre-adolescents (9–12 years old) and adolescents (13–18), Valencian region, Spain	Not reported	48 (Individual level)	6	Psychological well-being
Lacomba-Trejo 2020 [48]	Analyse the possible influence of self- esteem, peer problems and emotional competencies on anxiety- depressive clinical levels in adolescents with and without CD	Psychological/mental health	Not reported	FsQCA with SEM	Primary quantitative data (cross-sectional study)	Adolescents (12–18), Valencian region, Spain	Not reported	681 (individual level)	6	Anxiety- depressive clinical levels in adolescents with and without Chronic disease
Davis 2020 [6]	Provide actionable evidence to USAID and implementing organizations for strengthening their Orphan and Vulnerable Children programs	Physical health	"To identify the combinations of modifiable attributes that influenced knowledge of HIV status, a QCA was conducted. (46) this method recognizes that several different combinations of variables may lead to a particular outcome"	Sole FsQCA	Primary qualitative data (cross-sectional study)	70 activistas, 18 activista chefs, 12 supervisors, 6 CBO managers, 6 CBO monitoring and evaluation advisors, and the Covid project director, 3 provinces in Mozambique: Maputo, Gaza, Nampula	Not reported	70 (Individual level)	11	Percentage change in HIV knowledge status; percentage of beneficiaries with HIV status
McNamara 2015 [50]	Investigate the health impacts of a major trade liberalization episode in the textile and clothing sector: the phase-out of the Multi-Fibre Arrangement (MFA) in 2005	Maternal and infant mortality	"QCA techniques can be used for different purposes such as the testing of specific hypotheses, data exploration or for theoretical development. This study uses QCA primarily for theoretical development since existing theory surrounding trade liberalization, labour markets and health remains broad and imprecise"	QCA combined with process tracing methods	Secondary quantitative data (Case study)	32 countries (out of 53 countries) employment in the textile sector between 2000 and 2004	Not reported	32 (Country level)	5	Infant mortality rates
Blackman 2013 [39]	Identify conditions associated with the presence or absence of a narrowing gap in teenage pregnancy rates as measured by the differences between deprived local authority areas and the national average	Narrowing gap in teenage pregnancy	"The case-based method of Qualitative Comparative Analysis (QCA) is used to explore these data for possible causal pathways to different local teenage pregnancy rate outcomes"	CsQCA	Primary and secondary quantitative data (cross-sectional study)	27 local authority areas in England	Not reported	27 (local area level)	9	Narrowing gap between the area’s teenage pregnancy rate and the national average
Casaña‐Granell 2021	Analyse the adjustment of paediatric patients to their Short Stature diagnosis, and to determine the personal and family factors which influence and predict this adjustment process	psychological/mental health	"This methodology is particularly useful when there may be more than one explanation for a particular outcome, and they enable work with small samples”	FsQCA with hierarchical linear regression	Primary quantitative data (cross-sectional study)	101 adolescents diagnosed with idiopathic short stature (age 12–16), Valencian regions, Spain	Convenience sampling	101 (Individual level)	5	Anxiety-depression in adolescents
Gusap Coll 2020	Identify the importance of sociodemographic variables (sex and age), empathy, and EI on the psychological health of adolescents (self- esteem and satisfaction with life), comparing complementary methodologies, regression models, and fuzzy-set qualitative comparative analysis (fsQCA) models	Psychological/mental health	Not reported	FsQCA with linear regression model	Primary quantitative data (cross-sectional study)	991 adolescents from Spanish schools (Age 12–19), Valencian region, Spain	Convenience sampling	991 (Individual level)	8	Self-esteem; life satisfaction
Gilreath 2022 [58]	Explore the interplay between, and outcomes associated with, specific stressors related to relocation and deployment experiences among adolescent public-school students, and to determine key factors associated with maladaptive outcomes	Psychological/mental health	"To explore the interplay between, and outcomes associated with, specific stressors related to relocation and deployment experiences among adolescent public-school students, and to determine key factors associated with maladaptive outcomes"	CsQCA	Primary qualitative data (cross-sectional study)	24 military connected youth from middle and high schools near a military installation, Southern California	Not reported	24 (Individual level)	4	Maladaptive coping
Gusap Coll 2020	Identify the importance of empathy, emotional intelligence, and sociodemographic variables in at- attitudes towards tolerance of diversity and to compare complementary methodologies, regression models and fuzzy-set qualitative comparative analysis models (fsQCA)	Socio-emotional health	"Most of the studies have only used regression models in the adolescent context, con- temporary real-life case studies of modelling techniques on water quality, ignoring interactions and how different paths could lead to the same result, which could be assessed using qualitative comparative analysis models (QCA)"	FsQCA with regression analysis (Quant)	Primary quantitative data (cross-sectional study)	1069 adolescents (age 12–19). from six Spanish schools in the Valencian region, Spain	Convenience sampling	1069 (Individual level)	7	Tolerance to diversity in children and adolescents
Peris 2020	Estimate the combined contribution of body self-esteem (body satisfaction and physical attractiveness), personality traits (extraversion, neuroticism, disinhibition, and narcissism), and demographic factors (gender and age) in the prediction of four types of adolescent’s social networking and internet addiction (internet addiction symptoms, social media use, geek behaviour, and nomophobia)	Socio-emotional health	"Fuzzy-set qualitative comparative analysis (fsQCA) is a methodology that allows a more in-depth analysis of how a set of causal conditions contribute to a hypothesised outcome. QCA assumes that such outcome depends on a combination of different factors rather than on individual levels of those factors"	FSQCA with hierarchical linear multiple regression analysis	Primary quantitative data (cross-sectional study)	447 adolescents (age 13–16) from public and private high schools, Northern regions of Spain	Random probability sampling	447 (Individual level)	8	Social networking and internet addiction
Steven 2016 [54]	Explore the link between income inequality and adolescent cannabis use in the context of other social conditions at the national level in developed countries	Psychological/mental; education	QCA is used as an exploratory analysis of the ways in which the influences of social conditions may combine	Sole FsQCA	Secondary quantitative data (cross-sectional study)	22 European countries alongside the USA, Canada, and Australia	Not reported	22 (Country level)	6	Adolescent cannabis use
Van Kessel 2021 [55]	Synthesize the policy data of EDUCAUS and contribute to modelling pathways that are associated with the development of IE in EU Member States from the perspective of children with autism and identifying benchmarks that can be used to track the development of IE on a policy level	Education	"A Qualitative Comparative Analysis (QCA) is a suitable option to map the different possible pathways and interpret their meanings in light of existing literature”	sole QCA	Secondary quantitative data (cross-sectional study)	EU member states	Not reported	20 (Country level)	7	Inclusive education
Warren 2022 [15]	Elaborate and test whether the above hypotheses (generated from qualitative data) appear consonant with the pattern of contingencies found in fsQCA	Socio-emotional health	"In contrast to regression-based analysis, which examines statistical associations between multiple variables, QCA employs Boolean algebra (combinations of conditions linked by AND, NOT and OR) to examine what “pathways” (complex combinations of the presence or absence of factors) co-occur with certain outcome among a set of cases"	Sole FsQCA	Primary quantitative data (Randomized controlled trial)	40 mainstream state secondary schools in south-east England	Not reported	40 (School level)	4–6 (in each model)	Reduced bullying

### Participants

The research population consisted of individuals between the ages of [7–18] years. Many studies (n = 13) comprised children who were enrolled as elementary, secondary, and high school students attending public schools (see Table 1). Three studies recruited students from both public and private schools [41–43]. Two studies included pre-adolescent students [44, 45]. Two studies surveyed adult participants who are healthcare workers responsible for overseeing the health and well-being of children [6, 46]. Only a small number of research papers focused on the general adolescent population nationally (*n* = 2) [47, 48].

### Study design and study settings

**Fig. 2:**
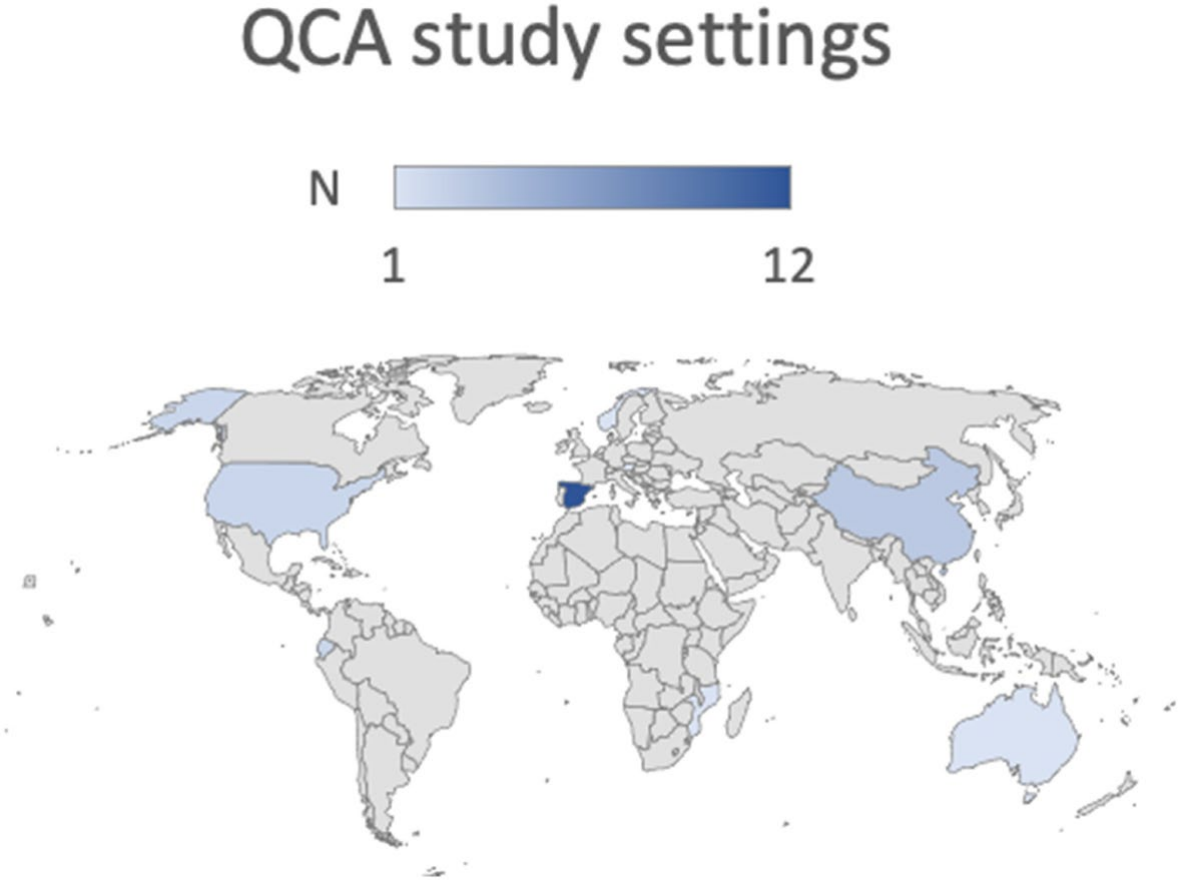
geographical distribution of study settings

Twenty-four of the included studies were cross-sectional (see Table 1), two were longitudinal studies [43, 49], two were case study [50] and randomized controlled trial [15]. The majority (*n* = 23) of studies were conducted predominantly in high-income countries. Country settings included: Spain (*n* = 12); England (*n* = 2); United States (*n* = 2); Austria (*n* = 1); Norway (*n* = 1); Australia (*n* = 1). Only three countries included were from low- and middle-income settings, which included Ecuador (*n* = 2) [5, 51], Mozambique (*n* = 1) [6] and China (*n* = 3) [44, 52, 53] (see Fig. 2 geographical distribution of study settings). Three studies involved multi-country settings, including a total of 32 countries [50], with 22 OECD countries [54] and 20 European member states [55] represented across these studies.

### Topics covered by child well-being studies

Most studies described topics related to children and adolescents, such as childhood adaptation in school contexts, smartphone use and disorders, emotional impact during COVID-19, lifestyle behaviours, depressive symptoms and anxiety in adolescence, and juvenile delinquency (*n* = 18) (see Table 1). Other studies (*n* = 9) examined interventions (e.g., language development) [46], health promotion programs (e.g., physical activity health promotion*)* [56] and policies (e.g., investigating maternal and infant mortality [50], reducing teenage pregnancy rates [39]). Studies assessed different components of well-being: psychological/mental health (*n* = 9); socio-emotional health (*n* = 7); physical and psychological/mental health (*n* = 3); physical health (*n* = 2); education (*n* = 2); multiple dimensions (*n* = 3) and objective well-being (*n* = 2) (see Table 1). The aims across studies differed and included studies looking at the combinations of predictors versus research examining combinations of outcomes. For example, Schoeps (2020) analysed the combined effect of parenting styles and psychopathological symptoms to predict personal, social, and school adaptation in school children [45]. Gao (2020) sought to understand predictors (e.g., life satisfaction, emotional stress, smart phone use disorder) that led to certain outcomes (e.g., maladaptation, mental illness) [44]. Herrera’s (2022) study, for example, examined the predictors of life satisfaction among adolescents in Ecuador during COVID-19 public health isolation measures [51].

### QCA application in child well-being studies

The case level of studies included the individual, classroom, school, and country levels. Three studies used countries as cases, ranging from 20 to 32 countries [50, 54, 55]. One study examined cases at the local area level [40], one study examined classrooms as cases (24 cases) [56], and one study examined schools as cases (40 cases) [15]. Data sources included primary quantitative data (*n* = 19) (see Table 1); secondary quantitative data (*n* = 4) [46, 50, 54, 55]; primary qualitative data (*n* = 4) [6, 52, 56, 57], primary and secondary quantitative data (*n* = 1) [39]; primary qualitative data, complemented by primary quantitative data driven from larger surveys (*n* = 1) [58]. Slightly over half of the studies relied on large sample sizes (from 50 to several thousand cases) (*n* = 16), while some studies included a small to medium sample size (with cases less than 50) (*n* = 12). The sample size of surveys used in studies ranged from 20–3047 individual participants (students and adolescents).

Although QCA was initially designed for small to medium cases [17], some studies in this review have combined QCA with other statistical analysis. In studies that took this approach it was framed as a complementary analysis to gain a better understanding of child well-being. For instance, half of the studies (*n* = 15) used QCA as a complementary analysis or to compare the findings from another form of statistical analysis, such as primarily regression modelling (*n* = 9) (see Table 1). The remaining studies used statistical analysis such as structural equation modelling (*n* = 5) [5, 48, 49, 51, 52] and process-tracing method (*n* = 1) [50]. QCA was used as a complementary method. For instance, Lacomba-Trejo (2020) compared the results of structural equation modeling with models based on QCA to analyse the possible influence of self-esteem, peer problems, and emotional competencies in adolescents [48]. One study used QCA to test an interpersonal-psychological theory of suicide among adolescents who attempted suicide [59]. QCA was also used as part of a randomised controlled trial on reducing school bullying [15]. Across the studies, QCA was often praised for its methodological ability to describe different causal pathways related to outcomes or combination of outcomes. For example, one study that applied QCA to predict life satisfaction identified sixteen paths that led to high levels of life satisfaction and eleven pathways were observed for low levels of life satisfaction.

Studies that used QCA alongside regression models (*n* = 9) included the same type of quantitative analysis/regression models (see Table 1). These studies used QCA to complement the results of their selected statistical analysis. The similarities between these studies are their shared statement about QCA’s ability to account for conjunctural causation in predicting the outcomes. Twelve of the studies in the review used QCA as a robust standalone analysis (see Table 1).

### Reported strengths for using QCA

Many studies (*n* = 9) observed that QCA is valuable for identifying various combinations and pathways to the desired outcome, drawing on terms such as "complexity," "equifinality” (different configurations that lead the same outcome), and "contextual conditions" (see Table 1). QCA was used for different stated purposes, including the testing of specific hypotheses, data exploration or for theoretical development (*n* = 2) [50, 58]. Additional reasons given by other studies (n = 1) included the use of QCA as an exploratory analysis of the ways in which the influences of social conditions may be used in combination [54]. In these cases, QCA examined the pathways (complex combinations of the presence or absence of conditions) that co-occur with certain outcomes among a set of cases. Other reported strengths included the use of Boolean algebra. Boolean algebra (including “and”, “or” and “not”) is set-theoretic comparative language for integrating qualitative and quantitative data, and can, for instance, be used as a tool to bring detailed qualitative interview data to enable comparative work within the dataset [58]. Interestingly, one of the reported benefits of using fsQCA is it can allow for the qualitative consideration of complex issues by bringing multiple pathways that lead to the outcome. This ability, according to Blackman (2013), “improves the robustness of case study research by using a systematic approach” [39].

There was consistent use of fsQCA (*n* = 24) across most included studies, which was justified because of its capacity to tackle complex social phenomenon (see Table 1). Authors highlighted that QCA can analyse the logical relationships between associate conditions and an outcome, providing more detailed results. For instance, a study which used both regression and fsQCA to assess emotional skills, self-esteem, and life-satisfaction indicated that fsQCA models provided more nuanced understanding of the pathways [4].

In addition, fsQCA offers an approach that includes qualitative and quantitative features, and helps identify specific types of cases for detailed within-case analyses [50]. Studies also reported that fsQCA enabled them to sustain the fine-grained differentiation of the conditions across cases [56]. Studies also demonstrated that QCA can be used for theory testing and/or theory development, for example, Gulbas et al. tested theory related to suicide among Latina adolescents [50]. Study authors reported specific strengths of fsQCA when it was used as a complementary analysis with other statistical analyses. As Liu (2022) suggested, fsQCA helps with asymmetrical and causal complexity analysis, therefore improving the identification of combined necessary and sufficient conditions for high or low level of juvenile delinquency [52]. Moreover, study authors explained that QCA provides greater horizonal complexity than regression analysis and it offers a more systematic way to analyse complex causality and logical relations between causal conditions and the outcomes [42]. Researchers indicated that a further strength of fsQCA models is that they are exploratory rather than confirmatory, offering a variety of pathways where predictors are combined in different ways [49].

### Reported weakness of the approach

Study authors discussed a number of limitations. One consensus was that findings cannot be generalised to other populations without doing cross-validation using further samples [42, 43, 48, 51, 53, 57]. Similarly, another fundamental constraint was the dependence on solely self-reported measures, which are not related to the method but to the data employed by individual studies [41, 43]. The number of conditions that can be included in QCA is another further limitation. Therefore, authors cautioned that there are several unmeasured conditions that could change the paths and could influence the coverage in the QCA model [60, 61].

The cited weaknesses also included the specific limitations of csQCA and fsQCA. A specific concern was related to the possibility of Type 1 error (false positives) and random error that make coincidental patterns which appear causal in fsQCA when it is applied to a relatively large sample size, as fsQCA is primarily developed for cases with small to medium sample sizes [44, 61]. A particular concern in the context of csQCA was the determination of thresholds for dichotomous conditions, as well as for continuous or categorical scales [39]. In the context of csQCA, a significant calibration challenge arises in relation to the interpretation of the value “0”; “Is it a true absence to non-mention” which cannot be fully triangulated [59]. In contrast to this, fsQCA is highly praised for its ability to account for more complexity and nuance between conditions and the outcomes. This capacity might be a reason fsQCA has become increasingly used in recent child well-being studies.

### Indicators of reporting quality

Of the all the studies, only two were found to satisfy all six reporting requirements [44, 46] When assessing the inclusion calibration of set membership, 10 studies provided full explanation, while 15 studies partially reported the calibration rules (see Table 2 for details). The remainder (*n* = 3) did not provide an explanation of the assignment of membership scores to their respective cases [52, 57, 62]. This is assessed whether the study discussed and justified the calibration of set membership scores being discussed in detail.

**Table 2:** Indicators of reporting quality

Publication details	Indicators of reporting quality					
Lead author	Calibration of set-membership scores discussed and justified	Evidence of familiarity with cases	Reporting truth-table	Reporting raw data matrix	Reporting solution formula	Reporting consistency and coverage measures
Schoeps	P	Y	N	N	Y	Y
Gao	Y	Y	Y	Y	Y	Y
Herrera	Y	Y	N	N	Y	Y
De la Barrera	P	Y	N	N	Y	Y
Coello	Y	Y	N	N	Y	Y
Gulbas	N	Y	Y	N	N	N
Liu	N	Y	N	N	Y	Y
Villanueva	Y	Y	N	N	Y	Y
Short, K	Y	Y	Y	Y	Y	Y
Mei, S	P	Y	N	N	Y	Y
Jimenez-Rodriguez	P	Y	N	N	Y	Y
Villanueva	P	Y	N	N	Y	Y
Wilhelmsen	P	Y	Y	N	Y	Y
Kien	Y	Y	Y	N	Y	Y
De la Barrera	P	Y	N	N	Y	Y
Valero-Moreno	P	Y	N	N	Y	Y
Lacomba-Trejo	P	Y	N	N	Y	Y
Davis	Y	Y	N	N	Y	Y
McNamara	Y	N	Y	Y	Y	Y
Blackman	Y	Y	Y	N	Y	N
Casaña‐Granell	N	Y	N	N	Y	N
Gusap Coll	P	Y	N	N	Y	Y
Gilreath	P	Y	N	Y	Y	N
Gusap Coll	P	Y	N	N	Y	Y
Peris	P	Y	N	N	Y	Y
Steven	Y	N	Y	N	Y	Y
Van Kessel	P	N	Y	N	Y	Y
Warren	P	Y	Y	N	Y	Y

Yes=Y, No=N and P=partially

‘Familiarity with cases’ was judged by examining each study's report on the population, the gender and age of the participants, and how well they knew the study sites. Most of the studies (*n* = 25) were considered adequate, while the remaining three were judged to be inadequate. Studies that used primary data (*n* = 25) were more likely to show that the researchers were familiar with the cases, while studies that used data from other sources indicated less familiarity. All studies (*n* = 25) that used primary data explained why they chose the cases they did. This was usually done with the selection criteria in mind, either to include a range of cases or to include cases with both presence and absence of outcomes.

Four studies reported raw data matrices in the paper or supplementary material; ten studies reported truth tables in the paper or supplementary material (see Table 2). Almost all studies (*n* = 27) reported solution formulas or pathways that lead to their outcome(s). Most studies (*n* = 21) reported the intermediate solution while providing explanation about the selection of it out of the three solutions, with some of them (*n* = 12) justifying it in relation to the literature. There are only a few studies which reported the parsimonious (*n* = 2) [39, 54] and complex solution (*n* = 1) [55]. The solution formula was provided as either direct output from the QCA (*n* = 3) and/or as a recipe in the text (*n* = 25). Most studies in the review (*n* = 25) reported at least some detail on the coverage (the number of cases with a particular configuration) and consistency (the percentage of similar causal configurations which result in the same outcome) (see Table 2).

## Discussion

Over the past decade, QCA has increasingly been used in child well-being research either as a standalone method or for complementary analyses combined with other methods. Our review builds on the literature of well-being that is viewed as a multi-dimensional concept, based on systematic reviews on child well-being conducted between 2003 and 2019 [8, 9]. The results of the scoping review suggested that scholars examining child well-being use multiple dimensions of objective and subjective well-being, which include well-being components including intellectual and school-related activities; family relationships, emotional support and social relationships; financial security; physical/psychological health and work safety; behaviours and risks/safety, housing, environment, and neighbourhood [9]. QCA studies in this review assessed different dimensions of well-being and included different outcomes of interest to measure child well-being. Outcomes addressed various physical, psychological, socio-emotional dimensions of child well-being. Our review indicated that studies to date have not included components of cognitive, social and economic dimensions. Future QCA work might also address these components as child well-being is now understood as a multi-dimensional concept.

Most studies in this review were conducted in high income countries, reflecting the disproportionate number of child well-being studies from the global north. Similarly, systematic reviews of QCA in public health research also show the dominance of research in HIC countries (both settings and author affiliations) [20]. Few studies have been conducted in low- and middle-income countries.

QCA techniques have their roots in the field of comparative policy studies. Rihoux et al.’s (2013) survey of peer-reviewed journal papers using QCA (*n* = 313) indicated that most were from the fields of political science and sociology, while less than 5% were from the health sciences [24]. Our review shows that there are increasing numbers of QCA studies published after 2015, indicating that the application of QCA in child well-being studies is relatively recent. While QCA was initially developed for small and medium N series of cases [1], our review demonstrates it is now often used with large quantitative data. Our review reveals a significant gap in the use of QCA for qualitative studies, as it is largely applied to studies with quantitative data sources, with fewer studies incorporating qualitative components or using qualitative data in a mixed method approach.

Notably, there is increasing interest in these methods as alternatives or complementary techniques to regression-oriented statistical methods for larger samples [42], such as surveys. For large sample sizes, QCA is often used alongside multiple regression analysis and structured equation modelling as was shown in our review. It is notable that these studies cite QCA as being more precise in the investigation of the complex pathways to the outcome(s). For example, in a study that assessed smart phone disorder, psychological characters, parent-adolescent relationship, and subjective quality of life among adolescents, authors showed that the results from the regression analysis demonstrated associations between risk factors and outcomes whereas the fsQCA results revealed configurations of all of the conditions that led to both the emergence of phone use disorder or not [44]. Another similar study which investigated psychological adjustment and subjective well-being in adolescents found that fsQCA models were able to include a great number of factors than regression models [49].

In the studies we reviewed, most studies share strengths of other QCA studies, such as its ability to identify different pathways (e.g., understanding the predictors of life satisfaction among adolescents; understanding predictors of emotional distress analysing the combined contribution of trait emotional intelligence, self-esteem, and perceived stress to pre-adolescents’ well-being) to the studies’ outcomes: subjective well-being; mental health. Authors often indicated that a key strength of using fsQCA was the emphasis on the precise measurements of the conditions and the outcome. For example, a study that investigated the combined effects of parenting styles and depression and anxiety symptoms to measure personal, social and school adaptation in school children used fsQCA to establish different combinations and in-depth descriptions of the outcome, which demonstrates its ability to talk about multiple data points and calibration of membership scores [45].

Despite these strengths, it seems that authors of QCA studies are reluctant to use QCA alone because of its emergence in the field, and, perhaps, because it has not yet been fully established as a method yet within the field. Moreover, QCA has shortcomings. For example, flawed calibration may lead to distorted findings [63]; pathways identified as effective can be coincidental rather than causal [61]; the number of conditions that can be effectively applied in QCA can affect the number of truth table rows significantly [64].

Although scoping reviews do not require the assessment of quality criteria, we assessed the inclusion of indicators for QCA studies by using best practices in QCA reporting. Schneider and Wagemann (2010) outlined good practices for QCA, offering foundational recommendations [28]. Building on this, Oana et al. (2021) updated and expanded these practices, incorporating recent methodological and technological developments and detailing various approaches to QCA [23]. More recent studies elaborated the good practices before, during, and after analytic moment in large-N QCA studies [65]. They also addresses key considerations such as cases and condition selection and provided guidance on appropriate ratio of cases to conditions [26, 66]. If used in line with good practices, QCA can be effectively employed as a stand-alone method, utilizing either qualitative or quantitative data [64].

### Strengths and limitations of the review

At present, there is no scoping review that looks at the utilisation of QCA in child/adolescents’ well-being research and interventions. Thus, our review contributes to an understanding of how QCA is being applied to measure the multi-dimensional nature of child well-being. This review has directly responded to this gap by producing an overview of the use of QCA by studies in the child well-being field, examining the existing use of QCA methods in LMIC and HIC countries by providing an assessment of how QCA is used in the field and compliance against reporting criteria.

Given the limited number of studies from LMICs child well-being and intervention studies, we expanded our inclusion criteria to incorporate studies from HICs. While this adjustment enhances comparative insights, it may introduce selection bias by incorporating findings from different socioeconomic contexts. All studies were not all double screened so accuracies may have arisen from this. In addition, restricting our search to publications in English may have caused us to miss bodies of work from non-English speaking countries, and excluding non-peer reviewed research and grey literature may have caused us to miss some examples of QCA methods in child well-being research.

## Conclusion

Our review is important methodologically for both QCA and child well-being fields of inquiry. QCA is an emerging method for the child health and well-being field, emerging, in part, for its methodological novelty for exploring causal complexities. This review has demonstrated the well-being dimensions (physical, psychological, and socio-emotional) in child well-being research that have been addressed to date using QCA. There is potential for QCA to be more widely used in child well-being research, to identify the different combinations of causal pathways to affect health and well-being of children. Outcomes in our included studies addressed various physical, psychological, socio-emotional dimensions of child well-being. Thus, future work would benefit from studying well-being components such as cognitive, social, and economic dimensions. As the data sources in this review are mostly quantitative, future work should look at using qualitative data as well, which can offer important in-depth data for case analysis.

In our review, there are a number of studies using QCA as a standalone method of analysis. QCA is also currently being used as a complementary analysis alongside statistical analysis. Our review reveals that it has potential to be used as a stand-alone QCA analysis if undertaken using good available practices. More comprehensive guidelines are now available that offer good practices to enhance the quality of the QCA research, more detailed reporting criteria for presenting the results, robustness check for transparency and replicability results. Thus, scholars are recommended to adhere to these good practices to establish the highest levels of transparency of the analysis. For the methodological development and credibility, we recommend that studies are reported with the usual attention to methodological transparency–including acknowledging its inherent weaknesses, and data availability, with key details that allow readers to judge the credibility of reported causal configurations.

## Supplementary Information

Supplementary Material 1.

Supplementary Material 2.

Supplementary Material 3.

Supplementary Material 4.

## Data Availability

Availability of data and materials Full search strategies and extraction forms are available by request from the first author. All data generated or analysed during the current study are included in this published article [and its supplementary information files].

## Abbreviations

COMPASS: Comparative methods for Systematic Cross-Case analysis
csQCA: Crisp set QCA
fsQCA: Fuzzy set QCA
mvQCA: Multivalue QCA
QCA: Qualitative Comparative Analysis
LMICs: Low– and Middle– income countries
HICs: High Income countries

## References

[1] Ragin C. Redesigning Social Inquiry: Fuzzy Sets and Beyond. Bibliovault OAI Repository, the University of Chicago Press. 2008.

[2] Burchett HED, Sutcliffe K, Melendez-Torres GJ, Rees R, Thomas J. Lifestyle weight management programmes for children: a systematic review using qualitative comparative analysis to identify critical pathways to effectiveness. Prev Med. 2018;106:1–12. 28865809 10.1016/j.ypmed.2017.08.025

[3] Chatterley C, Javernick-Will A, Linden KG, Alam K, Bottinelli L, Venkatesh M. A qualitative comparative analysis of well-managed school sanitation in Bangladesh. BMC Public Health. 2014;14(1):6. 24397540 10.1186/1471-2458-14-6 PMC3890631

[4] Guasp-Coll M, Navarro-Mateu D, Lacomba-Trejo L, Giménez-Espert MdC, Prado-Gascó VJ. Emotional skills in adolescents’ attitudes towards diversity: regression models vs qualitative comparative analysis models. Curr Psychol. 2021;41:8718–31.

[5] Coello MF, Valero-Moreno S, Herrera JS, Lacomba-Trejo L, Pérez-Marín M. Emotional impact in adolescents in Ecuador six months after the beginning of the COVID-19 pandemic. J Psychol. 2022;156(5):381–94. 35482962 10.1080/00223980.2022.2054921

[6] Davis A, Allen Z, Nascimento ND, Chapman J, Donco R, Velthausz D. A qualitative comparative analysis of the drivers of HIV status knowledge in orphans and vulnerable children in Mozambique. Glob Health Sci Pract. 2020;8(3):534–48. 33008862 10.9745/GHSP-D-20-00311 PMC7541122

[7] DiCenso A, Martin-Misener R, Bryant-Lukosius D, Bourgeault I, Kilpatrick K, Donald F, et al. Advanced practice nursing in Canada: overview of a decision support synthesis. Nurs Leadersh (Tor Ont). 2010;23 Spec No 2010:15–34. 10.12927/cjnl.2010.22267 21478685

[8] Pollard EL, Lee PD. Child well-being: a systematic review of the literature. Soc Indic Res. 2003;61(1):59–78.

[9] Cho EY-N, Yu F-Y. A review of measurement tools for child wellbeing. Children and Youth Services Review. 2020;119(C):S019074092031999X.

[10] Huppert FA. The State of Wellbeing Science. Wellbeing2014. p. 1–49.

[11] Spratt J. Conceptualising Wellbeing. In: Spratt J, editor. Wellbeing, Equity and Education: A Critical Analysis of Policy Discourses of Wellbeing in Schools. Cham: Springer International Publishing; 2017. p. 35–56.

[12] Suh E, Diener E, Fujita F. Events and subjective well-being: only recent events matter. J Pers Soc Psychol. 1996;70(5):1091–102. 8656337 10.1037//0022-3514.70.5.1091

[13] Ryan RM, Deci EL. On happiness and human potentials: a review of research on hedonic and eudaimonic well-being. Annu Rev Psychol. 2001;52:141–66. 11148302 10.1146/annurev.psych.52.1.141

[14] Prada A, Sanchez-Fernandez P. World child well-being index: a multidimensional perspective. Child Indic Res. 2021;14(6):2119–44.

[15] Warren E, Melendez-Torres GJ, Bonell C. Using fuzzy-set qualitative comparative analysis to explore causal pathways to reduced bullying in a whole-school intervention in a randomized controlled trial. J Sch Violence. 2022;21(4):381–96.

[16] Short K, Eadie P, Kemp L. Paths to language development in at risk children: a qualitative comparative analysis (QCA). BMC Pediatr. 2019;19(1):94. 30953552 10.1186/s12887-019-1449-z PMC6449893

[17] Ragin CC. Using qualitative comparative analysis to study causal complexity. Health Serv Res. 1999;34(5 Pt 2):1225–39. 10591281 PMC1089061

[18] Saridakis C, Zaefarian G, Ganotakis P, Angelidou S. A step-by-step guide of (fuzzy set) qualitative comparative analysis: from theory to practice via an implementation in a B2B context. Ind Mark Manage. 2022;107:92–107.

[19] Fiss P, Sharapov D, Cronqvist L. Opposites attract? Opportunities and challenges for integrating large-N QCA and econometric analysis. Polit Res Q. 2013;66:191–8.

[20] Hanckel B, Petticrew M, Thomas J, Green J. The use of Qualitative Comparative Analysis (QCA) to address causality in complex systems: a systematic review of research on public health interventions. BMC Public Health. 2021;21(877). 10.1186/s12889-021-10926-2 PMC8103124 33962595

[21] Forman-Hoffman VL, Middleton JC, McKeeman JL, Stambaugh LF, Christian RB, Gaynes BN, et al. Quality improvement, implementation, and dissemination strategies to improve mental health care for children and adolescents: a systematic review. Implement Sci. 2017. 10.1186/s13012-017-0626-4. 28738821 10.1186/s13012-017-0626-4 PMC5525230

[22] Melendez-Torres GJ, Sutcliffe K, Burchett HED, Rees R, Richardson M, Thomas J. Weight management programmes: re-analysis of a systematic review to identify pathways to effectiveness. Health Expect. 2018;21(3):574–84. 29508524 10.1111/hex.12667 PMC5980502

[23] Oana N, Schneider C, Thomann E. Qualitative Comparative Analysis Using R: A Beginner’s Guide2021.

[24] Rihoux B, Álamos-Concha P, Bol D, Marx A, Rezsohazy I. From niche to mainstream method? A comprehensive mapping of QCA application in journal articles from 1984 to 2011. Polit Res Q. 2013;66:175–84.

[25] Rihoux B, Ragin C. Configurational Comparative Methods: Qualitative Comparative Analysis (QCA) and Related Techniques. Thousand Oaks, California: SAGE Publications, Inc.; 2009. Available from: https://methods.sagepub.com/book/edvol/configurational-comparative-methods/toc.

[26] Glaesser J. Case-to-condition ratios in qualitative comparative analysis: adding cases instead of removing conditions. Field Methods. 2024. 10.1177/1525822X241231479.

[27] Legewie NM. An introduction to applied data analysis with qualitative comparative analysis. Forum Qual Soc Res. 2013;14:45.

[28] Schneider CQ, Wagemann C. Standards of good practice in qualitative comparative analysis (QCA) and fuzzy-sets. Comp Sociol. 2010;9:397–418.

[29] Glaesser J. Limited diversity and QCA solution types: assumptions and their consequences. Qual Quant. 2023;57(4):3485–97.

[30] Arksey H, O’Malley L. Scoping studies: towards a methodological framework. Int J Soc Res Methodol. 2005;8(1):19–32.

[31] Colquhoun HL, Levac D, O’Brien KK, Straus S, Tricco AC, Perrier L, et al. Scoping reviews: time for clarity in definition, methods, and reporting. J Clin Epidemiol. 2014;67(12):1291–4. 25034198 10.1016/j.jclinepi.2014.03.013

[32] Tricco AC, Lillie E, Zarin W, O’Brien KK, Colquhoun H, Levac D, et al. PRISMA extension for scoping reviews (PRISMA-ScR): checklist and explanation. Ann Intern Med. 2018;169(7):467–73. 30178033 10.7326/M18-0850

[33] Thongseiratch T, Leijten P, Melendez-Torres G. Online parent programs for children’s behavioral problems: a meta-analytic review. Eur Child Adolesc Psychiatry. 2020;29(11):1555–68. 31925545 10.1007/s00787-020-01472-0

[34] Moore DA, Russell AE, Matthews J, Ford TJ, Rogers M, Ukoumunne OC, et al. School-based interventions for attention-deficit/hyperactivity disorder: a systematic review with multiple synthesis methods. Rev Educ. 2018;6(3):209–63.

[35] Harris KM, Kneale D, Lasserson TJ, McDonald VM, Grigg J, Thomas J. School-based self-management interventions for asthma in children and adolescents: A mixed methods systematic review. Cochrane Database of Systematic Reviews. 2019;2019(1). 10.1002/14651858.CD011651.pub2 PMC6353176 30687940

[36] Thongseiratch T, Chalermphol K, Traipidok P, Charleowsak P. Promoting medication adherence in children with attention deficit hyperactivity disorder: a mixed-methods systematic review with meta-analysis and qualitative comparative analysis. J Atten Disord. 2024;28(2):139–50. 38006238 10.1177/10870547231211021

[37] Ouzzani M, Hammady H, Fedorowicz Z, Elmagarmid A. Rayyan—a web and mobile app for systematic reviews. Syst Rev. 2016;5(1): 210. 27919275 10.1186/s13643-016-0384-4 PMC5139140

[38] Pollock D, Peters MDJ, Khalil H, McInerney P, Alexander L, Tricco AC, et al. Recommendations for the extraction, analysis, and presentation of results in scoping reviews. JBI Evid Synth. 2023;21(3):520–32. 36081365 10.11124/JBIES-22-00123

[39] Blackman T. Exploring Explanations for Local Reductions in Teenage Pregnancy Rates in England: An Approach Using Qualitative Comparative Analysis. Soc Policy Soc. 2013;12(1):61–72. 24376371 10.1017/S1474746412000358 PMC3873005

[40] Blackman T, Wistow J, Byrne D. Using Qualitative Comparative Analysis to understand complex policy problems. Evaluation: The International Journal of Theory, Research and Practice. 2013;19(2):126–40.

[41] de la Barrera U, Schoeps K, Gil-Gómez JA, Montoya-Castilla I. Predicting adolescent adjustment and well-being: the interplay between socio-emotional and personal factors. Int J Environ Res Public Health. 2019. 10.3390/ijerph16234650. 31766641 10.3390/ijerph16234650 PMC6926821

[42] Villanueva L, Montoya-Castilla I, Prado-Gasco V. The importance of trait emotional intelligence and feelings in the prediction of perceived and biological stress in adolescents: hierarchical regressions and fsQCA models. Stress. 2017;20(4):355–62. 28595502 10.1080/10253890.2017.1340451

[43] Villanueva L, Prado-Gascó V, Montoya-Castilla I. Longitudinal analysis of subjective well-being in preadolescents: the role of emotional intelligence, self-esteem and perceived stress. J Health Psychol. 2022;27(2):278–91. 32830558 10.1177/1359105320951605

[44] Gao Q, Jia G, Fu E, Olufadi Y, Huang Y. A configurational investigation of smartphone use disorder among adolescents in three educational levels. Addict Behav. 2020. 10.1016/j.addbeh.2019.106231. 31862619 10.1016/j.addbeh.2019.106231

[45] Schoeps K, Valero-Moreno S, Perona AB, Pérez-Marín M, Montoya-Castilla I. Childhood adaptation: perception of the parenting style and the anxious-depressive symptomatology. J Spec Pediatr Nurs. 2020. 10.1111/jspn.12306. 32762136 10.1111/jspn.12306

[46] Short K, Eadie P, Kemp L. Paths to language development in at risk children: a qualitative comparative analysis (QCA). BMC Pediatr. 2019. 10.1186/s12887-019-1449-z. 30953552 10.1186/s12887-019-1449-z PMC6449893

[47] Casaña-Granell S, Lacomba-Trejo L, Montoya-Castilla I, Pérez-Marín M. Adolescence and short stature: factors in adjustment to the diagnosis. Qual Life Res. 2021;30(8):2275–86. 33665740 10.1007/s11136-021-02798-1

[48] Lacomba-Trejo L, Valero-Moreno S, Montoya-Castilla I, Pérez-Marín M. Psychosocial factors and chronic illness as predictors for anxiety and depression in adolescence. Front Psychol. 2020. 10.3389/fpsyg.2020.568941. 33071898 10.3389/fpsyg.2020.568941 PMC7530906

[49] Jimenez-Rodriguez T, De la Barrera U, Schoeps K, Valero-Moreno S, Montoya-Castilla I. Longitudinal analysis of adolescent adjustment: the role of attachment and emotional competence. Children (Basel). 2022;9(11): 08. 10.3390/children9111711 PMC9689061 36360439

[50] McNamara C. Trade liberalization, social policies and health: an empirical case study. Glob Health. 2015;11:19. 10.1186/s12992-015-0126-8 PMC4601122 26455360

[51] Herrera JS, Lacomba-Trejo L, Valero-Moreno S, Montoya-Castilla I, Pérez-Marín M. Do COVID-19 worries, resilience and emotional distress influence life satisfaction? Outcomes in adolescents in Ecuador during the pandemic: SEM vs. QCA. Children. 2022. 10.3390/children9030439. 35327811 10.3390/children9030439 PMC8947014

[52] Liu TH, Li SD. From family to peer systems: mixed-methods study of spillover mechanisms on juvenile delinquency in China. Crime Delinq. 2022. 10.1177/00111287221090957.

[53] Mei S, Lv J, Ren H, Guo X, Meng C, Fei J, et al. Lifestyle behaviors and depressive symptoms in Chinese adolescents using regression and fsQCA models. Front Public Health. 2022. 10.3389/fpubh.2022.825176. 35392470 10.3389/fpubh.2022.825176 PMC8980354

[54] Stevens A. Inequality and adolescent cannabis use: a qualitative comparative analysis of the link at national level. Drugs-Educ Prev Policy. 2016;23(5):410–21.

[55] Van Kessel R, Hrzic R, Cassidy S, Brayne C, Baron-Cohen S, Czabanowska K, et al. Inclusive education in the European Union: a fuzzy-set qualitative comparative analysis of education policy for autism. Soc Work Public Health. 2021;36(2):286–99. 33535919 10.1080/19371918.2021.1877590

[56] Kien C, Grillich L, Nussbaumer-Streit B, Schoberberger R. Pathways leading to success and non-success: a process evaluation of a cluster randomized physical activity health promotion program applying fuzzy-set qualitative comparative analysis. BMC Public Health. 2018. 10.1186/s12889-018-6284-x. 30563481 10.1186/s12889-018-6284-x PMC6299632

[57] Gulbas L, Szlyk H, Zayas LH. Evaluating the interpersonal-psychological theory of suicide among Latina adolescents using qualitative comparative analysis. Qual Psychol. 2019;6(3):297–311. 32051834 10.1037/qup0000131 PMC7015267

[58] Gilreath TD, Montiel Ishino FA, Sullivan KS, Okoror TA. Maladaptive coping among military-connected adolescents: Examining combined risk using QCA. Frontiers in Psychology. 2022;13. 10.3389/fpsyg.2022.948474 PMC9806339 36600698

[59] Gilreath TD, Ishino FMA, Sullivan KS, Okoror TA. Maladaptive coping among military-connected adolescents: examining combined risk using QCA. Front Psychol. 2022;13: 8. 10.3389/fpsyg.2022.948474 PMC9806339 36600698

[60] Short K, Eadie P, Kemp L. Influential factor combinations leading to language outcomes following a home visiting intervention: a qualitative comparative analysis (QCA). Int J Lang Commun Disord. 2020;55(6):936–54. 33051961 10.1111/1460-6984.12573

[61] Warren E, Melendez-Torres G, Bonell C. Using fuzzy-set qualitative comparative analysis to explore causal pathways to reduced bullying in a whole-school intervention in a randomized controlled trial. Journal of School Violence. 2022:No Pagination Specified.

[62] Casana-Granell S, Lacomba-Trejo L, Montoya-Castilla I, Perez-Marin M. Factors associated with stress when caring for a child with a short stature. Current Psychology: A Journal for Diverse Perspectives on Diverse Psychological Issues. 2021:No Pagination Specified.

[63] Fainshmidt S, Witt MA, Aguilera RV, Verbeke A. The contributions of qualitative comparative analysis (QCA) to international business research. J Int Bus Stud. 2020;51(4):455–66.

[64] Ide T, Mello PA. QCA in International Relations: a review of strengths, pitfalls, and empirical applications. Int Stud Rev. 2022. 10.1093/isr/viac008.

[65] Elgin DJ, Erickson E, Crews M, Kahwati LC, Kane HL. Applying qualitative comparative analysis in large-N studies: a scoping review of good practices before, during, and after the analytic moment. Qual Quant. 2024;58(5):4241–56.

[66] Mello P. Qualitative Comparative Analysis: An Introduction to Research Design and Application, Calibrating Sets (Chapter 5). 2021. p. 79–105.

