# Supplementary material for: The use of Qualitative Comparative Analysis (QCA) in child well-being research: a scoping review of research on child well-being research and interventions
Source: BMC Public Health. 2025 Sep 25;25:3122. doi: 10.1186/s12889-025-23821-x (PMC12462042; doi:10.1186/s12889-025-23821-x)
Supplement: Supplementary file 1 — Supplementary Material 1. [file 12889_2025_23821_MOESM1_ESM.pdf]

The use of Qualitative Comparative Analysis (QCA) in child well-being research: a scoping review of research on child well-being research and interventions in low- and middle- income countries.

Aye Thiri Kyaw[1], Meghna Ranganathan[1], Cathy Zimmerman[1], Isabelle Pearson[1], Emily Warren[2], Benjamin Hanckel[3]

[1]Gender Violence and Health Centre, Department of Global Health, and Development, LSHTM, London, UK.

[2]Department of Health Services Research and Policy, LSHTM, London, UK

[3]Institute for Culture and Society, Western Sydney University, Sydney, Australia.

Corresponding author: Aye Thiri Kyaw (aye-thiri.kyaw@lshtm.ac.uk)

Background

Qualitative Comparative Analysis (QCA) is a method that can analyse several different causal conditions (causal complexity) to our outcome of interest. here is a growing literature using QCA to measure child well-being and evaluate interventions in low-and middle-income settings (1, 2, 3, 4, 5). The authors in this field consistently noted the need for understanding and making sense of the causal complexity of well-being as an outcome (5, 6, 7). To our knowledge, there has not been any extensive review of the QCA literature on child well-being in low-income and middle-income countries (LMICs), despite the increasing use of QCA in these settings. Our scoping review aims to map and examine the application of QCA in child well-being research and interventions in LMICs. It will also assess the potential of QCA as a method for its usefulness in assessing causal complexity in child well-being research and evaluations. assessing child well-being.

The World Health Organisation (WHO) defines “well-being” as “a positive state experienced by individuals and societies. Like health, it is a resource for daily life and is determined by social, economic, and environmental conditions”. However, it is a broad concept that has been operationalised in varied ways across child well-being studies (8, 9). Despite the variations of its use, most researchers now agree that “well-being is a multidimensional construct incorporating mental/psychological, physical and social dimensions that affects all aspects of children/adolescents lives”(9). In the child well-being literature, one common way to conceptualise child well-being is to classify it into objective and subjective well-being (10). Objective well-being relates to external conditions to the individual, such as income, literacy and life expectancy, which can be assessed using a variety of validated indicators (11). Subjective well-being, in contrast, refers to an individual’s perception and experience of life (11, 12). Subjective aspect of well-being is classified by its two main historical roots: hedonic well-being and eudaimonic well-being. Hedonic well-being emphasizes happiness, positive and negative emotions, and life satisfaction. In contrast, the eudaimonic well-being emphasizes experience of personal functioning and the pursuit of meaningful goals and self-actualization (13).

The current literature on children’s well-being acknowledges that children’s needs are multidimensional (14). For instance, in their systematic review of published child well-being studies between 1991 and 1999, Pollard and Lee (2003) identified five distinct dimensions of well-being: physical, social, psychological, cognitive and economic as the important areas contributing to a child’s overall well-being and development (8). Indicators of child well-being for the physical dimension include physical activities, wellness knowledge, and eating attitudes; indicators for the psychological dimension include depression, emotions, mental health, or illness; indicators for the cognitive dimension include intellectual and school-related activities; indicators for the social dimension include family relationships, emotional support,

socially desirable behaviours, and communication skills; and indicators for the economic and financial dimension include financial security (8). A more recent review of measurement tools for child well-being studies from 2000 to 2019 elaborated on this work, proposing well-being dimensions include physical health and safety; behaviours and risks/safety, housing, environment, and neighbourhood; social relationships, psychological health and socio-emotional well-being. This also connects to WHO definition that well-being is determined by social, economic, and environmental conditions. In this way, wellbeing is a multidimensional concept (see Figure 1). In this scoping review, we will attempt to cover all the studies that use the QCA to examine the different dimensions of child well-being.

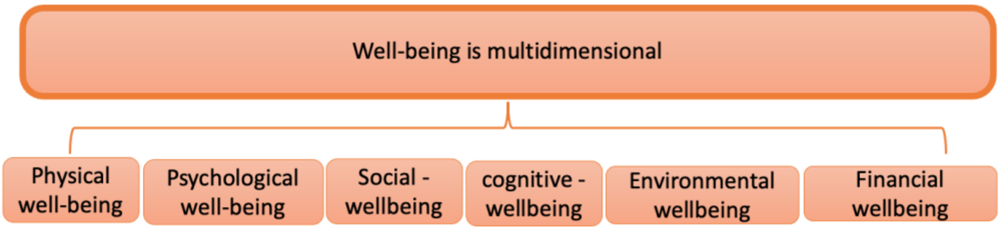

Figure 1 shows multiple dimensions of child well-being from the literature.

Child well-being and Qualitative Comparative Analysis

One approach that is increasingly being used to examine child wellbeing is QCA (1, 5, 15, 16). QCA is a case-based approach that explores relationships between 'conditions' and 'outcomes. QCA identifies the combinations of conditions (often referred to as pathways or recipes) that lead to an outcome or absence of an outcome. One of the strengths of QCA is its ability to explore equifinality, or the different combinations of causal conditions which may lead to the same outcome; (6). For instance, wellbeing might be the outcome of interest – a QCA enables a researcher to examine 'conditions' (or 'casual recipes') that may lead to wellbeing; QCA suits small to medium number of cases (10-50 cases) although there has been recent QCA applications in studies with larger sample sizes (17, 18). QCA brings the qualitative and

quantitative approaches together (6). It is a mixed methods approach and can use both qualitative and quantitative data sources (19). QCA requires the familiarity with cases for in-depth knowledge like qualitative research approach. It looks for empirical patterns in and across the cases like quantitative research approach. QCA uses the principle of set theoretic methods , which is a method that defines the cases by assigning set membership based on theoretical and conceptual guidance (20). Its aim is to investigate the causal relationships between ‘conditions’ and ‘outcomes’ of the membership sets.

There are two main variants of QCA: crisp-set QCA (csQCA) and fuzzy-set QCA (fsQCA). There is a further variant called multi-value QCA (mvQCA) (21). CsQCA is exclusively used in conventional sets where cases are given dichotomous values such as 0 or 1, either in or out of the membership sets. However, partial memberships are permitted in fsQCA capturing different dimensions of the empirical manifestations that inform and interpret the analysis (6). A central element of QCA is the identification of configurations of necessary and sufficient conditions to the outcomes between (complex) sets (6, 20, 22). For a condition to be necessary, it must be always present when the outcome is present. Without a necessary condition, the outcome will not be present. In contrast, a condition is sufficient when the condition is present, yet the outcome could still result from other conditions. Whenever set-theory methods are used, as is done in QCA, it is also possible to identify conditions that alone are not necessary and sufficient but important for an outcome to occur. They are called INUS and SUIN conditions (20). INUS stands for “insufficient but necessary part of a condition which is itself unnecessary but sufficient for the result” (23, 24). SUIN stands for a “sufficient, but unnecessary part of a factor that is insufficient, but necessary for the result” (20).In most QCA studies, as Legewie argues, conditions or combinations of conditions are ‘quasi-necessary’ or ‘quasi-sufficient’ for the outcome (25).

In QCA, one of the key tools in examining causal complexity is called the truth table (6). The truth table lists all the logically possible conditions designated by the researcher and permits that different recipes may lead to the presence or absence of the outcome (26). The aim of truth table is to examine whether the cases with specific causal conditions share the same outcome. However, all the empirical information is limited in their variation in real life. It is called limited diversity (6). The truth table can identify the issue of limited diversity by showing the empty rows. These empty rows are called logical remainders in QCA. FsQCA software can look the logical remainders and the configurations that display the outcome. This step is called Boolean minimization. QCA uses three basic Boolean operations called Intersection (logical AND), Union (logical OR), Negation (logical NOT). Logical AND "*" is used to examine the membership score of the case in the combinations of conditions. (25, 27).

While QCA has been used to assess child well-being, to our knowledge no review on its usefulness has been published. Therefore, we seek to do a scoping review on the use of QCA in child-well-being studies and assess how the method has been used to measure the large, complex, and heterogeneous nature of child well-being studies.

Method

Scope reviews are a type of knowledge synthesis that responds to an exploratory research question by systematically searching, selecting, and synthesising existing knowledge in order to map the key concepts, types of evidence, and research gaps related to a defined field or area (28, 29). Scoping reviews are becoming more prevalent and widely used in a variety of disciplines (30).

The proposed scoping review will follow the Arksey and O'Malley framework, which has been a methodological guidance for the scoping reviews since its formal introduction in 2014. The framework includes five key phases: (1) identifying the research question, (2) identifying relevant studies, (3) study selection, (4) charting the data, and (5) collating, summarising, and reporting the results; and an optional consultation. Colquhoun et al enhanced the framework by increasing the clarity and rigor of the scoping review process (29).We have already completed the first step – identifying the research question. In this review, 'consultation exercise' will not be conducted as it goes beyond the scope of the review.

Framework stage 1: Identifying the research question.

The primary aim of this scoping review is to examine how QCA has been used in measuring dimensions of well-being and interventions in low-and middle-income countries?

The proposed scoping review will answer the following research questions:

1. What are the different ways of using QCA in child well-being research and interventions? evaluating interventions and assessing dimensions of well-being in LMICs?
2. What data sources have been used in QCAs?
3. What additional analyses are being done along with QCA by the researchers?
4. What are the self-reported strengths, and limitations of QCA approaches across this literature identified by researchers who have used QCA? And how is it different from the existing QCA guidance?
5. What are the existing gaps and what implications do these have for future child well-being research and interventions?

Framework stage 2: identifying relevant studies.

Data sources and Search strategy

We developed a search strategy that combined search terms for "Qualitative Comparative Analysis", "Child" and "Well-Being". In total, seven electronic databases (Embase, Psyinfo, Medline, Social Policy and Practice, Global Health Scopus, and Web of Science) will be searched. To be as comprehensive as possible, additional searches will be further made from different sources: reference lists, hand-searching of key journals and using existing networks such as www.compass.org, which is the main repository of QCA literature.

Framework stage 3: study selection

The studies will be downloaded into Endnote for deduplication. The remaining studies will be uploaded to Rayyan for abstract screening. After the deduplication and abstract screening,

To determine the relevance of studies found through the search, a two-stage screening method will be used. For the first level of screening, only the title and abstract of the research will be assessed. Then all relevant papers will be found. If the papers are not available at LSHTM, we will look for them at the libraries of University of London. Second reviewer and LSHTM PhD student, Isabelle Pearson, will be responsible for double screening for 10% of the included studies. During the full text screening, each study will be categorised as include, exclude and unsure with reasons. The disagreements will be resolved during the discussion and will get consultation from the third reviewer. A PRISMA flow chart will be used for the study selection process.

Inclusion criteria

	Inclusion criteria	
Type of participants	Children under 18 years old.	
Countries	low- and middle-income countries	
Type of sources:	Any studies that employ Qualitative Comparative Analysis – crisp-set QCA, Fuzzy set QCA or multi-value QCA to report one or more dimensions of child well-being.	
Type of publications	Peer-reviewed	
Timespan	From 2000 to 2023	
Unit of analysis	Individual, interventions that address well-being as an outcome of interest.	
Type of outcomes	Any study that is assessing child well-being as an outcome. Well-being is multidimensional in nature. Therefore, we note that the varied outcomes might be included.	

	Dimensions of child well-being are generally concerned with the areas:<br>- Health/physical health and safety.<br>- Education.<br>- Economic and material well-being.<br>- Behaviours and risks/safety.<br>- Housing, environment, and neighbourhood.<br>- Social relationships.<br>- Psychological health and socio-emotional wellbeing as well as subjective well-being, which usually covered self-defined health, well-being at school, and personal well-being.<br>- Objective well-being<br>- capability	
Language	English	

Framework stage 4: Charting the data (data extraction)

The following data will be extracted using a standardized format endorsed by all the reviewers to minimise the discrepancies. An Excel workbook will be created to include the following data: this will be an iterative process. The table will be continually updated until the review team is becoming familiar with the sources in the review.

Type of data to be extracted	
1. Lead author	
2. Publication date	
3. Country/region setting	
4. Aim of the study	
5. Rationale for using QCA	
6. Type of QCA (Crisp or Fuzzy Set or MV?)	
7. Study population	
8. Number of cases	
9. Case level of QCA	
10. Data used for analysis-source (primary data or secondary analysis)	
11. Type of data (qual/quant/both)	
12. Outcomes and details of these (e.g., how measures	

Framework stage five: Collating, Summarizing and Reporting the Results

The reporting of the results will align with the checklist of preferred reporting items for systematic reviews and meta-analyses extension for Scoping Reviews (PRISMA-ScR). Data from the included studies will be presented in the form of a descriptive numerical summary and qualitative analysis of the data. The descriptive numeracy summary will explain study characteristics, rationale of using QCA and type of QCA, number of cases and case level of the included studies' results. The summary will be produced as map or in tabulated form . During the analysis of the results, I will use Nvivo, which is a qualitative data organizational software, to analyse the results. for narrative synthesis.

Quality assessment of the included studies

Currently, scoping reviews do not include a formal assessment of quality of the included studies (28). However, there is some available guidance as to how to best report QCA (22, 31, 32)The following criteria used by Hacknel et at in their systematic review of QCA on public health interventions will be used to assess the quality of the study (22):

i) Whether the study shows the familiarity of the cases.

ii) Whether the study reports an explicit and detailed justification for selection of cases.

iii) Whether the study report the truth table.

iv) Whether the study analysis contains the solution formula; and

v) Whether the consistency and coverage measures are reported.

Strength and limitation

At present, there is no scoping review that looks at the utilisation of QCA in child/adolescents' well-being research and interventions. The review will contribute to an understanding of how QCA is applied to measure the multi-dimensional nature of child well-being. This review will respond to this gap by producing an overview of the use of QCA and researchers in the child well-being field using QCA methods in low-and middle-income countries. It will also provide an assessment of how QCA is used in the field and its compliance with reporting criteria by the researchers. This review is limited to the peer reviewed articles written in English. Therefore, it is likely to underrepresent the high-quality research written in other languages.

Funding

This study is funded by Millby-Council for At-Risk Academics (CARA) scholarship. The funders will play no part in the conducting or reporting of the scoping review.

Reference

1. Burchett HED, Sutcliffe K, Melendez-Torres GJ, Rees R, Thomas J. Lifestyle weight management programmes for children: A systematic review using Qualitative Comparative Analysis to identify critical pathways to effectiveness. Prev Med. 2018;106:1-12.
2. Chatterley C, Javernick-Will A, Linden KG, Alam K, Bottinelli L, Venkatesh M. A qualitative comparative analysis of well-managed school sanitation in Bangladesh. BMC Public Health. 2014;14(1):6.
3. Guasp-Coll M, Navarro-Mateu D, Lacomba-Trejo L, Giménez-Espert MdC, Prado-Gascó VJ. Emotional skills in adolescents' attitudes towards diversity: Regression models vs qualitative comparative analysis models. Current Psychology. 2021;41:8718 - 31.
4. Coello MF, Valero-Moreno S, Herrera JS, Lacomba-Trejo L, Pérez-Marín M. Emotional Impact in Adolescents in Ecuador Six Months after the Beginning of the COVID-19 Pandemic. J Psychol. 2022;156(5):381-94.
5. Davis A, Allen Z, Nascimento ND, Chapman J, Donco R, Velthausz D. A Qualitative Comparative Analysis of the Drivers of HIV Status Knowledge in Orphans and Vulnerable Children in Mozambique. Glob Health Sci Pract. 2020;8(3):534-48.
6. Ragin C. Redesigning Social Inquiry: Fuzzy Sets and Beyond. Bibliovault OAI Repository, the University of Chicago Press. 2008.
7. DiCenso A, Martin-Misener R, Bryant-Lukosius D, Bourgeault I, Kilpatrick K, Donald F, et al. Advanced practice nursing in Canada: overview of a decision support synthesis. Nurs Leadersh (Tor Ont). 2010;23 Spec No 2010:15-34.

8. Pollard EL, Lee PD. Child Well-being: A Systematic Review of the Literature. Social Indicators Research. 2003;61(1):59-78.
9. Cho EY-N, Yu F-Y. A review of measurement tools for child wellbeing. Children and Youth Services Review. 2020;119(C):S019074092031999X.
10. Huppert FA. The State of Wellbeing Science. Wellbeing2014. p. 1-49.
11. Spratt J. Conceptualising Wellbeing. In: Spratt J, editor. Wellbeing, Equity and Education: A Critical Analysis of Policy Discourses of Wellbeing in Schools. Cham: Springer International Publishing; 2017. p. 35-56.
12. Suh E, Diener E, Fujita F. Events and subjective well-being: only recent events matter. J Pers Soc Psychol. 1996;70(5):1091-102.
13. Ryan RM, Deci EL. On happiness and human potentials: a review of research on hedonic and eudaimonic well-being. Annu Rev Psychol. 2001;52:141-66.
14. Prada A, Sanchez-Fernandez P. World Child Well-Being Index: A Multidimensional Perspective. Child Indicators Research. 2021;14(6):2119-44.
15. Warren E, Melendez-Torres GJ, Bonell C. Using fuzzy-set qualitative comparative analysis to explore causal pathways to reduced bullying in a whole-school intervention in a randomized controlled trial. Journal of School Violence. 2022;21(4):381-96.
16. Short K, Eadie P, Kemp L. Paths to language development in at risk children: a qualitative comparative analysis (QCA). BMC Pediatrics. 2019;19(1):94.
17. Greckhamer T, Misangyi VF, Fiss PC, editors. Chapter 3 The Two QCAs: From a Small-N to a Large-N Set Theoretic Approach2013.
18. Blackman T. Exploring Explanations for Local Reductions in Teenage Pregnancy Rates in England: An Approach Using Qualitative Comparative Analysis. Soc Policy Soc. 2013;12(1):61-72.
19. Kane H, Lewis MA, Williams PA, Kahwati LC. Using qualitative comparative analysis to understand and quantify translation and implementation. Transl Behav Med. 2014;4(2):201-8.
20. Schneider CQ, Wagemann C. Set-Theoretic Methods for the Social Sciences: A Guide to Qualitative Comparative Analysis: Cambridge University Press; 2012.
21. Vink MP, Vliet Ov. Potentials and Pitfalls of Multi-value QCA:Response to Thiem. Field Methods. 2013;25(2):208-13.
22. Hanckel B, Petticrew M, Thomas J, Green J. The use of Qualitative Comparative Analysis (QCA) to address causality in complex systems: a systematic review of research on public health interventions. BMC Public Health. 2021;21(1):877.
23. Ide T, Mello PA. QCA in International Relations: A Review of Strengths, Pitfalls, and Empirical Applications. International Studies Review. 2022;24(1).
24. Mackie JLi. Causes and Conditions. American Philosophical Quarterly. 1965;2(4):245 - 64.
25. Legewie NM. An Introduction to Applied Data Analysis with Qualitative Comparative Analysis. Forum Qualitative Social Research. 2013;14:45.
26. Ragin CC. Using qualitative comparative analysis to study causal complexity. Health Serv Res. 1999;34(5 Pt 2):1225-39.
27. Mackie JL. Causes and Conditions. American Philosophical Quarterly. 1965;2(4):245 - 64.
28. Arksey H, O'Malley L. Scoping studies: towards a methodological framework. International Journal of Social Research Methodology. 2005;8(1):19-32.
29. Colquhoun HL, Levac D, O'Brien KK, Straus S, Tricco AC, Perrier L, et al. Scoping reviews: time for clarity in definition, methods, and reporting. J Clin Epidemiol. 2014;67(12):1291-4.

30. Anderson S, Allen P, Peckham S, Goodwin N. Asking the right questions: Scoping studies in the commissioning of research on the organisation and delivery of health services. Health Research Policy and Systems. 2008;6(1):7.
31. Schneider CQ, Wagemann C. Standards of Good Practice in Qualitative Comparative Analysis (QCA) and Fuzzy-Sets. Comparative Sociology. 2010;9:397-418.
32. Rihoux B, Álamos-Concha P, Bol D, Marx A, Rezsohazy I. From Niche to Mainstream Method? A comprehensive mapping of QCA application in journal articles from 1984 to 2011. Political Research Quarterly. 2013;66:175-84.
