# Supplementary material for: The use of Qualitative Comparative Analysis (QCA) in child well-being research: a scoping review of research on child well-being research and interventions
Source: BMC Public Health. 2025 Sep 25;25:3122. doi: 10.1186/s12889-025-23821-x (PMC12462042; doi:10.1186/s12889-025-23821-x)
Supplement: Supplementary file 2 — Supplementary Material 2. [file 12889_2025_23821_MOESM2_ESM.pdf]

Supplementary file 1

Search results

Search results were uploaded to EndNote X9 and deduplicated. Number of results pre- and post- deduplication are listed in the table below.

Database name	Endnote import order	Number of references before deduplication	Number of references	After deduplication (auto)	Manual deduplicate
Medline	1	104	117	33	30
Embase	2	110	124	71	64
Global Health	3	20	21	11	10
Psyinfo	4	135	157	124	118
Scopus	5	67	72	69	55
Web of Science	6	101	123	86	73
Social policy	7		9	7	4
Econlit	8		3	3	3
Total			626	404 (222-auto)	357 (47)

3. Search strategies

3.1 OvidSP Medline

Database name	Medline
Database platform	OvidSP
Dates of database coverage	1946 -2022
Data searched	1 September 2022
Searched by	ATK
Number of hits	104

1 (Qualitative Comparative Analysis or Crisp set Qualitative Comparative Analysis or Fuzzy set Qualitative Comparative Analysis or QCA or csQCA or fsQCA or multi-value QCA).mp. [mp=title, book title, abstract, original title, name of substance word, subject heading word, floating sub-heading word, keyword heading word, organism supplementary concept word, protocol supplementary concept word, rare disease supplementary concept word, unique identifier, synonyms] 1537

2 exp Child/ 2097138

3 child*.mp. 2674744

4 exp Child, Preschool/ 979957

5 preschool child*.mp. 15627

6 exp Infant/ 1227786

7 infan*.mp. 1388609

8 exp Infant, Newborn/ 658930

9	infant newborn.mp.	655192
10	exp Adolescent/	2186907
11	Adolescent*.mp.	2258232
12	exp Young Adult/	996579
13	young adult.mp.	1021027
14	exp Minors/	2761
15	minor*.mp.	354316
16	boy*.mp.	170475
17	girl*.mp.	165939
18	orphan*.mp.	23293
19	adopted child*.mp.	1009
20	fostered child*.mp.	23
21	teen*.mp.	34408
22	juvenile.mp.	93044
23	son*.mp.	199155
24	daughter*.mp.	28509
25	youth.mp.	88766
26	school age.mp.	15704
27	young person.mp.	1280
28	young people.mp.	33882
29	exp Child Labor/	71
30	or/2-29	5405002
31	1 and 30	118
32	well-being.mp.	102024
33	exp Health/	420920
34	health.mp.	3437567
35	exp Adolescent Health/	1764
36	adolescent health.mp.	13195
37	exp Child Health/	4581
38	child health.mp.	52037
39	exp Infant Health/	1195
40	infant health.mp.	4520
41	exp Mental Health/	55335
42	mental health.mp.	246276
43	exp Oral Health/	19413
44	oral health.mp.	38128
45	exp Public Health/	8932288
46	public health.mp.	400353
47	exp "Social Determinants of Health"/	5784
48	social determinants of health.mp.	12486
49	physical health.mp.	27285
50	exp Child Development/	65626
51	child development.mp.	62252
52	exp Language Development/	17763
53	language development.mp.	20884
54	exp Child Language/	5027
55	child* language.mp.	6215

56	exp Mental Competency/	8574
57	mental competency.mp.	8618
58	exp Motivation/	188782
59	motivation.mp.	132507
60	exp Safety/	87718
61	safety.mp.	693959
62	psychological development.mp.	1168
63	emotional development.mp.	2070
64	social development.mp.	4945
65	educational development.mp.	473
66	exp Social Behavior/	285901
67	social behavio?r.mp.	69190
68	exp Aggression/	42861
69	aggression.mp.	52407
70	exp Altruism/	7522
71	altruism.mp.	9786
72	exp Competitive Behavior/	8511
73	competitive behavio?r.mp.	8828
74	exp Cooperative Behavior/	45718
75	cooperative behavio?r.mp.	46697
76	exp Deception/	5512
77	decepti*.mp.	10070
78	dehumanization.mp.	987
79	exp Empowerment/	695
80	empowerment.mp.	16106
81	exp Sexual Harassment/	2129
82	sexual harassment.mp.	3137
83	exp Help-Seeking Behavior/	1124
84	help seeking behavio?r.mp.	2601
85	exp Helping Behavior/	3240
86	helping behavio?r.mp.	3570
87	exp Incivility/	263
88	incivil*.mp.	781
89	exp Permissiveness/	239
90	permissive*.mp.	22494
91	exp Prejudice/	35652
92	exp Self-Control/	5066
93	self-control.mp.	9195
94	exp Harassment, Non-Sexual/	6236
95	non-sexual harassment.mp.	2
96	exp Shyness/	630
97	shy*.mp.	3814
98	exp Social Adjustment/	23613
99	social adjustment.mp.	25022
100	exp Social Conformity/	3167
101	social conformity.mp.	3305
102	exp Social Desirability/	4449

103	social desirability.mp.	6152
104	exp Social Discrimination/	11466
105	social discrimination.mp.	2617
106	exp Psychological Distance/	3258
107	psychological distance.mp.	3525
108	exp Social Dominance/	7067
109	social dominance.mp.	5688
110	exp Social Isolation/	21490
111	social isolation.mp.	23159
112	exp Social Marginalization/	575
113	social marginali#ation.mp.	851
114	exp Social Skills/	2539
115	social skills.mp.	7761
116	exp Social Stigma/	11891
117	social stigma.mp.	13589
118	exp Stereotyping/	11845
119	stereotyp*.mp.	41334
120	exp Educational Status/	58079
121	education* status.mp.	61677
122	educational achievement.mp.	1344
123	cognitive development.mp.	8038
124	disabil*.mp.	322702
125	exp Mortality/	419592
126	mortality.mp.	1335578
127	exp Morbidity/	633360
128	morbidity.mp.	444199
129	Student Dropouts/	1877
130	student dropout*.mp.	1893
131	dropout*.mp.	21909
132	exp Pregnancy in Adolescence/	8384
133	pregnancy in adolescence.mp.	8461
134	exp Crime/	151866
135	crim*.mp.	74119
136	drug.mp.	6209559
137	cigarette.mp.	66573
138	exp Depression/	143489
139	depressi*.mp.	534596
140	exp Suicide/	71841
141	suicid*.mp.	108767
142	exp Suicide, Attempted/	22194
143	attempted suicid*.mp.	6151
144	school engagement.mp.	435
145	intellectual development.mp.	1349
146	exp Child Behavior Disorders/	20756
147	child behavio?r disorder*.mp.	20780
148	exp Character/	2866
149	character.mp.	83399

150 identity.mp. 176830
151 personal life.mp. 1446
152 confidence.mp. 632013
153 exp Empathy/ 22026
154 empathy.mp. 30851
155 civic life.mp. 126
156 connect*.mp. 506727
157 competen*.mp. 260925
158 nutrition*.mp. 445057
159 exp Obesity/ 248615
160 obes*.mp. 417457
161 exp Vaccines/ 264646
162 vaccine*.mp. 374799
163 adolescent pregnancy.mp. 2876
164 teen* pregnancy.mp. 2639
165 wellness.mp. 13382
166 human flourishing.mp. 209
167 exp personal satisfaction/ 23119
168 personal satisfaction.mp. 23752
169 life satisfaction.mp. 10039
170 social wellbeing.mp. 481
171 spiritual wellbeing.mp. 158
172 physical wellbeing.mp. 462
173 intellectual wellbeing.mp. 0
174 occupational wellbeing.mp. 38
175 financial wellbeing.mp. 94
176 environmental wellbeing.mp. 12
177 capab*.mp. [mp=title, book title, abstract, original title, name of substance word, subject heading word, floating sub-heading word, keyword heading word, organism supplementary concept word, protocol supplementary concept word, rare disease supplementary concept word, unique identifier, synonyms] 462053
178 exp "Quality of Life"/ 249079
179 quality of life.mp. 419926
180 or/32-179 17357669
181 1 and 31 and 180 104

Ovid MEDLINE(R) ALL <1946 to May 31, 2023>

1 (Qualitative Comparative Analysis or Crisp set Qualitative Comparative Analysis or Fuzzy set Qualitative Comparative Analysis or QCA or csQCA or fsQCA or multi-value QCA).mp. [mp=title, book title, abstract, original title, name of substance word, subject heading word, floating sub-heading word, keyword heading word, organism supplementary concept word, protocol supplementary concept word, rare disease supplementary concept word, unique identifier, synonyms, population supplementary concept word, anatomy supplementary concept word] 1683
2 exp Child/ 2144489
3 child*.mp. 2746510

4	exp Child, Preschool/	988382
5	preschool child*.mp.	16248
6	exp Infant/	1248405
7	infan*.mp.	1415991
8	exp Infant, Newborn/	671530
9	exp Adolescent/	2212050
10	adolescent*.mp.	2288878
11	Young Adult/	1009535
12	young adult.mp.	1034732
13	exp Minors/	2808
14	minor*.mp.	368935
15	boy*.mp.	175842
16	girl*.mp.	171801
17	orphan*.mp.	24116
18	adopted child*.mp.	1029
19	fostered child*.mp.	25
20	teen*.mp.	35673
21	juvenile.mp.	96030
22	son*.mp.	206955
23	daughter*.mp.	29244
24	youth.mp.	94453
25	school age.mp.	16389
26	young person.mp.	1352
27	young people.mp.	36220
28	Child Labor/	79
29	child labor.mp. or Child Labor/	531
30	or/2-29	5538324
31	1 and 30	132
32	well-being.mp.	111405
33	Health/	25047
34	health.mp.	3612943
35	exp Adolescent Health/	1877
36	adolescent health.mp.	13711
37	exp Child Health/	4981
38	child health.mp.	54192
39	exp Infant Health/	1259
40	infant health.mp.	4816
41	exp Mental Health/	60647
42	mental health.mp.	266025
43	exp Oral Health/	20205
44	oral health.mp.	40266
45	exp Public Health/	9197252
46	public health.mp.	426244
47	physical health.mp.	29616
48	exp Child Development/	66349
49	child development.mp.	63213
50	exp Language Development/	18068

51	language development.mp.	21479
52	exp Child Language/	5075
53	child* language.mp.	6372
54	exp Mental Competency/	8602
55	mental competency.mp.	8650
56	exp Motivation/	193348
57	motivation.mp.	139142
58	exp Safety/	88662
59	safety.mp.	739680
60	psychological development.mp.	1225
61	emotional development.mp.	2235
62	social development.mp.	5281
63	educational development.mp.	508
64	exp Social Behavior/	291974
65	social behavio?r.mp.	70548
66	exp Aggression/	44051
67	aggression.mp.	53834
68	exp Altruism/	7721
69	altruism.mp.	10163
70	exp Competitive Behavior/	8537
71	competitive behavio?r.mp.	8870
72	exp Cooperative Behavior/	45969
73	cooperative behavio?r.mp.	46986
74	exp Deception/	5654
75	decepti*.mp.	10425
76	exp Empowerment/	744
77	empowerment.mp.	17434
78	agency.mp.	64193
79	exp Sexual Harassment/	2241
80	sexual harassment.mp.	3341
81	Help-Seeking Behavior/	1172
82	help-seeking behavio?r.mp.	2747
83	exp Helping Behavior/	3267
84	helping behavio?r.mp.	3628
85	exp Incivility/	305
86	incivil*.mp.	843
87	exp Pessimism/	378
88	pessimis*.mp.	4474
89	exp Self-Control/	5613
90	self-control.mp.	9647
91	exp Shyness/	641
92	shy*.mp.	3966
93	exp Social Adjustment/	23662
94	social adjustment.mp.	25132
95	exp Social Conformity/	3178
96	social conformity.mp.	3321
97	exp Social Desirability/	4458

98	social desirability.mp.	6261
99	exp Social Discrimination/	12523
100	social discrimination.mp.	2705
101	exp Psychological Distance/	3270
102	psychological distance.mp.	3571
103	exp Psychological Distress/	6699
104	psychological distress.mp.	29376
105	exp Social Dominance/	7119
106	social dominance.mp.	5775
107	exp Social Isolation/	25421
108	social isolation.mp.	24449
109	exp Social Marginalization/	585
110	social marginalization.mp.	837
111	exp Anxiety/	110762
112	anxiety.mp.	306881
113	exp Social Skills/	2658
114	social skills.mp.	8165
115	exp Social Stigma/	12695
116	social stigma.mp.	14583
117	exp Stereotyping/	12035
118	stereotyp*.mp.	42595
119	educational achievement.mp.	1393
120	cognitive development.mp.	8638
121	exp Mortality/	423018
122	mortality.mp.	1389528
123	exp Morbidity/	646947
124	morbidity.mp.	463823
125	exp Student Dropouts/	1889
126	dropout*.mp.	22823
127	dropout*.mp.	22823
128	exp Pregnancy in Adolescence/	8473
129	pregnancy in adolescence.mp.	8555
130	exp Crime/	156201
131	crime.mp.	40503
132	drug.mp.	6341631
133	exp Depression/	149769
134	depressi*.mp.	559856
135	exp Suicide/	74286
136	suicid*.mp.	113522
137	exp Suicide, Attempted/	22800
138	attempted suicid*.mp.	6336
139	school engagement.mp.	474
140	intellectual development.mp.	1406
141	behavior disorders.mp.	29819
142	confidence.mp.	672697
143	exp Empathy/	22725
144	empathy.mp.	32429

145 connect*.mp. 534230
146 competen*.mp. 269837
147 nutrition*.mp. 465930
148 exp Obesity/ 258792
149 obes*.mp. 437828
150 exp Pregnancy in Adolescence/ 8473
151 teen* pregnancy.mp. 2729
152 exp Vaccines/ 275821
153 vaccine*.mp. 396864
154 wellness.mp. 14545
155 flourishing.mp. 2502
156 exp Personal Satisfaction/ 24318
157 personal satisfaction.mp. 24838
158 personal satisfaction.mp. 24838
159 life satisfaction.mp. 10922
160 spiritual well-being.mp. 1585
161 social well-being.mp. 2556
162 physical well-being.mp. 3109
163 intellectual well-being.mp. 5
164 occupational well-being.mp. 170
165 financial well-being.mp. 412
166 environmental well-being.mp. 45
167 economic well-being.mp. 740
168 capability.mp. 135840
169 exp "Quality of Life"/ 266302
170 quality of life.mp. 446183
171 or/32-170 17529300
172 1 and 31 and 171 115

3.3 OvidSP Embase

Database name	Embase
Database platform	OvidSP
Dates of database coverage	1947-2022
Data searched	1 September 2022
Searched by	ATK
Number of hits	110

Embase Classic+Embase <1947 to 2022 August 31>

1 (Qualitative Comparative Analysis or Crisp set Qualitative Comparative Analysis or Fuzzy set Qualitative Comparative Analysis or QCA or csQCA or fsQCA or multi-value QCA).mp. [mp=title, abstract, heading word, drug trade name, original title, device manufacturer, drug manufacturer, device trade name, keyword heading word, floating subheading word, candidate term word] 2889
2 exp child/ 3340377
3 child*.mp. 3332829

4	exp preschool child/	677642
5	preschool child*.mp.	682000
6	exp infant/	1263209
7	infan*.mp.	1154147
8	exp newborn/	669163
9	newborn.mp.	796748
10	newborn infant.mp.	10579
11	exp adolescent/	1834522
12	adolescent*.mp.	1922588
13	exp young adult/	471562
14	young adult.mp.	503210
15	exp "minor (person)"/	834
16	minor*.mp.	467223
17	exp boy/	46071
18	boy*.mp.	253219
19	exp girl/	58271
20	girl*.mp.	243536
21	orphan*.mp.	33646
22	adopted child*.mp.	1588
23	fostered child*.mp.	33
24	teen*.mp.	48421
25	exp juvenile/	4331343
26	juvenile.mp.	172675
27	exp son/	5221
28	son*.mp.	370124
29	exp daughter/	6138
30	daughter*.mp.	39256
31	youth.mp.	105663
32	school age.mp.	22020
33	young person.mp.	2257
34	young people.mp.	47806
35	exp child labor/	290
36	child labor.mp.	636
37	exp childhood/	124068
38	childhood.mp.	502997
39	or/2-38	6268531
40	1 and 39	152
41	exp wellbeing/	109404
42	wellbeing.mp.	101633
43	exp psychological well-being/	26283
44	psychological wellbeing.mp.	3955
45	exp health/	849936
46	health.mp.	4879339
47	exp adolescent health/	10282
48	adolescent health.mp.	16364
49	exp child health/	3se3285
50	child* health.mp.	101729

51	infant health.mp.	4054
52	exp mental health/	210733
53	mental health.mp.	371804
54	oral health.mp.	34437
55	exp public health/	226536
56	public health.mp.	561608
57	physical health.mp.	35949
58	exp child development/	52445
59	child development.mp.	58388
60	exp language development/	18355
61	language development.mp.	21109
62	child* language.mp.	1856
63	exp mental capacity/	99996
64	mental capacity.mp.	18590
65	mental competency.mp.	161
66	exp motivation/	164546
67	motivation.mp.	166252
68	exp safety/	548370
69	safety.mp.	1366419
70	psychological development.mp.	1791
71	exp mental development/	45206
72	mental development.mp.	8483
73	emotional development.mp.	2834
74	social development.mp.	4858
75	educational development.mp.	559
76	exp social behavior/	2077064
77	social behavio?r.mp.	102007
78	exp aggression/	128191
79	agression.mp.	677
80	exp altruism/	8262
81	altruism.mp.	9756
82	exp competitive behavior/	6356
83	competitive behavio?r.mp.	6642
84	exp cooperation/	66226
85	cooperation.mp.	175738
86	cooperative behavio?r.mp.	1566
87	exp deception/	3142
88	decepti*.mp.	9088
89	exp dehumanization/	838
90	dehumanization.mp.	1060
91	exp empowerment/	12003
92	empowerment.mp.	23736
93	exp sexual harassment/	3675
94	sexual harassment.mp.	4236
95	help seeking behavior/	14413
96	help seeking behavio?r.mp.	15340
97	helping behavior.mp.	437

98	exp incivility/	410
99	incivil*.mp.	835
100	exp attitude/	893628
101	attitude.mp.	525471
102	exp prejudice/	3823
103	prejudice.mp.	9105
104	non-sexual harassment/	71
105	non-sexual harassment.mp.	72
106	exp shyness/	420
107	shy*.mp.	12392
108	exp social adaptation/	143910
109	social adaptation.mp.	26339
110	social adjustment.mp.	3949
111	social conformity.mp.	269
112	exp social desirability/	5036
113	social desirability.mp.	6407
114	exp social discrimination/	29288
115	social discrimination.mp.	5674
116	exp psychological distance/	40
117	psychological distance.mp.	349
118	exp social dominance/	6707
119	social dominance.mp.	7068
120	exp social isolation/	31289
121	social isolation.mp.	35748
122	exp social exclusion/	2423
123	social exclusion.mp.	4088
124	social marginalization.mp.	303
125	exp social competence/	6683
126	social competence.mp.	8694
127	social skills.mp.	8411
128	exp social stigma/	12851
129	social stigma.mp.	14054
130	exp stereotyping/	2820
131	stereotyp*.mp.	42619
132	exp educational status/	119136
133	education* status.mp.	100930
134	educational achievement.mp.	1695
135	exp cognitive development/	8937
136	cognitive development.mp.	14530
137	exp disability/	190724
138	disabil*.mp.	409773
139	exp mortality/	1341566
140	mortality.mp.	1832844
141	exp morbidity/	421883
142	morbidity.mp.	781550
143	exp school dropout/	566
144	student dropout*.mp.	60

145	school dropout/	566
146	school dropout.mp.	1038
147	dropout*.mp.	20528
148	exp adolescent pregnancy/	10199
149	adolescent pregnancy.mp.	10684
150	exp crime/	102873
151	crim*.mp.	96089
152	exp drug/	3459315
153	drug.mp.	12943053
154	exp cigarette/	3921
155	cigarette.mp.	126773
156	exp depression/	574030
157	depressi*.mp.	851239
158	exp suicide/	66957
159	suicid*.mp.	156911
160	suicide attempt/	37539
161	suicid* attempt.mp.	39112
162	school engagement.mp.	418
163	exp academic achievement/	42445
164	academic achievement.mp.	43207
165	intellectual development.mp.	2083
166	exp behavior disorder/	488678
167	behavio?r disorder*.mp.	71965
168	exp character/	239913
169	character.mp.	109252
170	exp character disorder/	781
171	character disorder.mp.	849
172	exp identity/	53036
173	identity.mp.	199434
174	personal life.mp.	1977
175	confidence.mp.	790750
176	exp empathy/	30887
177	empathy.mp.	36053
178	civic life.mp.	147
179	connect*.mp.	680704
180	exp competence/	134282
181	competen*.mp.	284321
182	exp nutrition/	2697159
183	nutrition*.mp.	621738
184	exp obesity/	621941
185	obes*.mp.	699043
186	vaccine*.mp.	509159
187	teen* pregnancy.mp.	3314
188	wellness.mp.	18577
189	human flourishing.mp.	189
190	exp happiness/	11376
191	happiness.mp.	15031

192 exp satisfaction/ 279315
193 satisfaction.mp. 338914
194 personal satisfaction.mp. 1138
195 exp life satisfaction/ 11563
196 life satisfaction.mp. 16527
197 exp social well-being/ 1293
198 social wellbeing.mp. 864
199 exp spiritual well-being/ 306
200 spiritual wellbeing.mp. 345
201 exp physical well-being/ 3770
202 physical wellbeing.mp. 892
203 intellectual wellbeing.mp. 3
204 occupational wellbeing.mp. 36
205 economic well-being/ 253
206 economic wellbeing.mp. 139
207 financial wellbeing.mp. 124
208 environmental wellbeing.mp. 18
209 capab*.mp. 555517
210 exp "quality of life"/ 595140
211 quality of life.mp. 726629
212 or/41-211 23213913
213 40 and 212 110

Embase Classic+Embase <1947 to 2023 May 31> (2 June 2023)

1 (Qualitative Comparative Analysis or Crisp set Qualitative Comparative Analysis or Fuzzy set Qualitative Comparative Analysis or QCA or csQCA or fsQCA or multi-value QCA).mp. [mp=title, abstract, heading word, drug trade name, original title, device manufacturer, drug manufacturer, device trade name, keyword heading word, floating subheading word, candidate term word] 3076
2 exp child/ 3507996
3 child*.mp. 3506342
4 exp preschool child/ 698334
5 preschool child*.mp. 703087
6 exp infant/ 1314757
7 infan*.mp. 1204109
8 exp newborn/ 693722
9 newborn.mp. 826897
10 newborn infant.mp. 10749
11 exp adolescent/ 1905171
12 adolescent*.mp. 1998881
13 exp young adult/ 508793
14 young adult.mp. 541486
15 exp "minor (person)"/902
16 minor*.mp. 494076
17 exp boy/ 46888

18	boy*.mp.	264977
19	exp girl/	58971
20	girl*.mp.	255572
21	orphan*.mp.	36157
22	adopted child*.mp.	1652
23	fostered child*.mp.	34
24	teen*.mp.	51084
25	exp juvenile/	4536346
26	juvenile.mp.	184463
27	exp son/	5750
28	son*.mp.	384537
29	exp daughter/	6288
30	daughter*.mp.	40964
31	youth.mp.	115226
32	school age.mp.	23300
33	young person.mp.	2449
34	young people.mp.	52109
35	exp child labor/	338
36	child labor.mp.	686
37	exp childhood/	134508
38	childhood.mp.	531412
39	or/2-38	6580418
40	1 and 39	170
41	exp wellbeing/	123956
42	wellbeing.mp.	113583
43	exp psychological well-being/	30269
44	psychological wellbeing.mp.	4474
45	exp health/	918374
46	health.mp.	5232856
47	exp adolescent health/	11028
48	adolescent health.mp.	17377
49	exp child health/	36553
50	child* health.mp.	107710
51	infant health.mp.	4465
52	exp mental health/	237838
53	mental health.mp.	409368
54	oral health.mp.	37005
55	exp public health/	249567
56	public health.mp.	610461
57	physical health.mp.	39725
58	exp child development/	53843
59	child development.mp.	60135
60	exp language development/	19035
61	language development.mp.	21950
62	child* language.mp.	1978
63	exp mental capacity/	107346
64	mental capacity.mp.	20418

65	mental competency.mp.	172
66	exp motivation/	175550
67	motivation.mp.	176236
68	exp safety/	571756
69	safety.mp.	1477105
70	psychological development.mp.	1898
71	exp mental development/	47591
72	mental development.mp.	8755
73	emotional development.mp.	3063
74	social development.mp.	5299
75	educational development.mp.	608
76	exp social behavior/	2215079
77	social behavio?r.mp.	106011
78	exp aggression/	135912
79	agression.mp.	703
80	exp altruism/	8613
81	altruism.mp.	10158
82	exp competitive behavior/	6400
83	competitive behavio?r.mp.	6689
84	exp cooperation/	68014
85	cooperation.mp.	181124
86	cooperative behavio?r.mp.	1655
87	exp deception/	3345
88	decepti*.mp.	9560
89	exp dehumanization/	915
90	dehumanization.mp.	1148
91	exp empowerment/	13333
92	empowerment.mp.	25890
93	exp sexual harassment/	3996
94	sexual harassment.mp.	4578
95	help seeking behavior/	15519
96	help seeking behavio?r.mp.	16473
97	helping behavior.mp.	475
98	exp incivility/	484
99	incivil*.mp.	925
100	exp attitude/	937245
101	attitude.mp.	542131
102	exp prejudice/	4198
103	prejudice.mp.	9652
104	exp shyness/	509
105	shy*.mp.	13272
106	exp social adaptation/	152568
107	social adaptation.mp.	26740
108	social adjustment.mp.	4098
109	social conformity.mp.	285
110	exp social desirability/	5132
111	social desirability.mp.	6616

112	exp social discrimination/	34040
113	social discrimination.mp.	6104
114	exp psychological distance/	68
115	psychological distance.mp.	392
116	exp social dominance/	6802
117	social dominance.mp.	7176
118	exp social isolation/	34114
119	social isolation.mp.	39079
120	exp social exclusion/	2803
121	social exclusion.mp.	4503
122	social marginalization.mp.	331
123	exp social competence/	7582
124	social competence.mp.	9623
125	social skills.mp.	8902
126	exp social stigma/	14628
127	social stigma.mp.	15784
128	exp stereotyping/	3004
129	stereotyp*.mp.	44893
130	exp educational status/	134008
131	education* status.mp.	111878
132	educational achievement.mp.	1774
133	exp cognitive development/	9843
134	cognitive development.mp.	15776
135	exp disability/	203590
136	disabil*.mp.	440765
137	exp mortality/	1444681
138	mortality.mp.	1976542
139	exp morbidity/	448664
140	morbidity.mp.	833226
141	exp school dropout/	627
142	student dropout*.mp.	64
143	school dropout/	627
144	school dropout.mp.	1119
145	dropout*.mp.	22375
146	exp adolescent pregnancy/	10468
147	adolescent pregnancy.mp.	10977
148	exp crime/	107874
149	crim*.mp.	100279
150	exp drug/	3620241
151	drug.mp.	13551685
152	exp depression/	625244
153	depressi*.mp.	912380
154	exp suicide/	70309
155	suicid*.mp.	167304
156	suicide attempt/	40136
157	suicid* attempt.mp.	41836
158	school engagement.mp.	465

159	exp academic achievement/	44480
160	academic achievement.mp.	45098
161	intellectual development.mp.	2187
162	exp behavior disorder/	521827
163	behavio?r disorder*.mp.	75040
164	exp character disorder/	787
165	character disorder.mp.	855
166	exp identity/	56139
167	identity.mp.	212911
168	confidence.mp.	870523
169	exp empathy/	32745
170	empathy.mp.	38251
171	connect*.mp.	731460
172	exp competence/	136533
173	competen*.mp.	298282
174	exp nutrition/	2862747
175	nutrition*.mp.	659843
176	exp obesity/	670414
177	obes*.mp.	752465
178	vaccine*.mp.	553193
179	teen* pregnancy.mp.	3462
180	wellness.mp.	20544
181	human flourishing.mp.	219
182	exp happiness/	12307
183	happiness.mp.	16188
184	exp satisfaction/	298638
185	satisfaction.mp.	362538
186	personal satisfaction.mp.	1242
187	exp life satisfaction/	12548
188	life satisfaction.mp.	17823
189	exp social well-being/	2111
190	social wellbeing.mp.	981
191	exp spiritual well-being/	471
192	spiritual wellbeing.mp.	374
193	exp physical well-being/	4630
194	physical wellbeing.mp.	1020
195	intellectual wellbeing.mp.	3
196	occupational wellbeing.mp.	41
197	economic well-being/	431
198	economic wellbeing.mp.	156
199	financial wellbeing.mp.	152
200	environmental wellbeing.mp.	21
201	capab*.mp.	594037
202	exp "quality of life"/	648225
203	quality of life.mp.	792430
204	exp anxiety/	300322
205	anxiety.mp.	505771

206 distress.mp. 302390
207 or/41-206 24424154
208 1 and 39 and 207 124

3.4 OvidSP Global Health

Database name	Global Health
Database platform	OvidSP
Dates of database coverage	1910-2022
Data searched	1 September 2022
Searched by	ATK
Number of hits	20<br>21
Note*	Use embase filter for global health for importing endnote

1 (Qualitative Comparative Analysis or Crisp set Qualitative Comparative Analysis or Fuzzy set Qualitative Comparative Analysis or QCA or csQCA or fsQCA or multi-value QCA).mp. [mp=abstract, title, original title, heading words, cabicodes words] 164
2 exp children/ 423611
3 child*.mp. 514675
4 exp preschool children/ 16143
5 preschool child*.mp. 18036
6 exp infants/ 161236
7 infan*.mp. 190599
8 newborn.mp. 29385
9 exp adolescents/ 80025
10 adolescent*.mp. 91845
11 exp young adults/ 15576
12 young adult*.mp. 31633
13 minor*.mp. 88994
14 exp boys/ 28597
15 boy*.mp. 56570
16 exp girls/ 35534
17 girl*.mp. 58018
18 exp orphans/ 881
19 orphan*.mp. 3223
20 adopted child*.mp. 267
21 fostered child*.mp. 139
22 teen*.mp. 82554
23 juvenile.mp. 8717
24 exp sons/ 252
25 son*.mp. 20675
26 exp daughters/ 463
27 daughter*.mp. 4707
28 exp youth/ 22315
29 youth.mp. 31260

30	school age.mp.	5622
31	young person.mp.	164
32	young people.mp.	10818
33	child labour.sh.	398
34	child labo?r.mp.	489
35	childhood.mp.	64652
36	or/2-35	785838
37	well-being.mp.	21297
38	psychological wellbeing.mp.	433
39	exp health/	426306
40	health.mp.	1386503
41	adolescent health.mp.	2168
42	exp child health/	15162
43	child* health.mp.	20277
44	infant health.mp.	1659
45	exp mental health/	34503
46	mental health.mp.	45397
47	exp oral health/	5571
48	oral health.mp.	10050
49	exp public health/	182228
50	public health.mp.	374971
51	social determinants of health.mp.	3070
52	physical health.mp.	6079
53	exp child development/	3533
54	child* development.mp.	5542
55	language development.mp.	375
56	child* language.mp.	88
57	mental capacity.mp.	124
58	exp mental ability/	30278
59	mental ability.mp.	15772
60	mental competency.mp.	7
61	exp motivation/	5635
62	motivation.mp.	14356
63	exp safety/	167193
64	safety.mp.	285394
65	psychological development.mp.	166
66	exp cognitive development/	3870
67	cognitive development.mp.	4494
68	mental development.mp.	4287
69	exp emotional development/	174
70	emotional development.mp.	484
71	exp social development/	455
72	social development.mp.	1617
73	educational development.mp.	89
74	social behaviour.sh.	3324
75	social behavio?r.mp.	3809
76	exp aggression/	1122

77 aggression.mp. 3082
78 exp altruism/ 103
79 altruism.mp. 380
80 competitive behavio?r.mp. 36
81 exp cooperation/ 1229
82 cooperation.mp. 11235
83 cooperative behavio?r.mp. 39
84 deception.mp. 201
85 dehumani#ation.mp. [mp=abstract, title, original title, heading words, cabicodes words] 41
86 exp empowerment/ 6240
87 empowerment.mp. 6800
88 exp sexual harassment/ 174
89 sexual harassment.mp. 394
90 help seeking behavio?r.mp. 298
91 helping behavio?r.mp. 21
92 incivility.mp. 31
93 exp attitudes/ 81791
94 attitude*.mp. 94690
95 prejudice.mp. 1023
96 self control.mp. 1106
97 non-sexual harassment.mp. 0
98 shy*.mp. 476
99 social adaptation.mp. 115
100 exp social adjustment/ 60
101 social adjustment.mp. 198
102 social conformity.mp. 26
103 social desirability.mp. 401
104 social discrimination.mp. 129
105 psychological distance.mp. 23
106 exp social dominance/ 45
107 social dominance.mp. 97
108 exp social isolation/ 852
109 social isolation.mp. 2370
110 social exclusion.mp. 714
111 social marginali#ation.mp. [mp=abstract, title, original title, heading words, cabicodes words] 146
112 social competence.mp. 197
113 social skills.mp. 548
114 exp social stigma/ 6001
115 social stigma.mp. 6287
116 stereotyp*.mp. 2095
117 educational status.mp. 2464
118 educational achievement.mp. 347
119 exp academic achievement/ 2227
120 academic achievement.mp. 2596
121 exp disabilities/ 13427

122	disabilit*.mp.	31091
123	exp mortality/	188032
124	mortality.mp.	258862
125	exp morbidity/	40892
126	morbidity.mp.	88765
127	school dropout*.mp.	261
128	dropout*.mp.	1887
129	exp student dropouts/	104
130	student dropout*.mp.	106
131	pregnant adolescents.sh.	1007
132	pregnant adolescent*.mp.	1300
133	adolescent pregnancy.mp.	518
134	teen* pregnancy.mp.	804
135	exp crime/	5480
136	crim*.mp.	9166
137	exp drugs/	940094
138	exp drugs/	940094
139	drug*.mp.	1250935
140	exp drug abuse/	22183
141	drug abuse.mp.	23313
142	exp cigarettes/	20670
143	cigarette*.mp.	31752
144	exp smoking/	1617
145	smoking.mp.	95481
146	exp depression/	31119
147	depressi*.mp.	57125
148	suicide.sh.	9815
149	suicid*.mp.	13096
150	suicid* attempt.mp.	1364
151	school engagement.mp.	82
152	intellectual development.mp.	300
153	behavio?r disorder.mp.	105
154	character.mp.	13578
155	character disorder.mp.	2
156	identity.mp.	22959
157	personal life.mp.	219
158	confidence.mp.	158896
159	empathy.mp.	997
160	civic life.mp.	18
161	connect*.mp.	47659
162	competen*.mp. [mp=abstract, title, original title, heading words, cabicodes words]	23359
163	exp nutrition/	105450
164	nutrition*.mp.	1170352
165	exp obesity/	146801
166	obes*.mp.	172344
167	vaccine*.mp.	149748

168 exp wellness/ 7434
169 wellness.mp. 9500
170 exp longevity/ 4806
171 longevity.mp. 8050
172 human flourishing.mp. 32
173 happiness.mp. 1379
174 exp enjoyment/ 552
175 enjoyment.mp. 2027
176 satisfaction.mp. 19611
177 personal satisfaction.mp. 230
178 exp work satisfaction/ 1947
179 work satisfaction.mp. 1993
180 life satisfaction.mp. 1548
181 social well-being.mp. 596
182 spiritual wellbeing.mp. 23
183 physical wellbeing.mp. 113
184 occupational wellbeing.mp. 5
185 economic wellbeing.mp. 72
186 financial wellbeing.mp. 21
187 environmental wellbeing.mp. 7
188 capability.mp. 14064
189 exp "quality of life"/ 26806
190 quality of life.mp. 41734
191 or/37-190 3575899
192 1 and 36 and 191 20

3.5 Ovid SP Psyinfo

Database name	Psyinfo
Database platform	OvidSP
Dates of database coverage	1806-2022
Data searched	1 September 2022
Searched by	ATK
Number of hits	135

1 (Qualitative Comparative Analysis or Crisp set Qualitative Comparative Analysis or Fuzzy set Qualitative Comparative Analysis or QCA or csQCA or fsQCA or multi-value QCA).mp. [mp=title, abstract, heading word, table of contents, key concepts, original title, tests & measures, mesh word] 822
2 child*.mp. 875260
3 pre-school child*.mp. 1666
4 infan*.mp. 137045
5 newborn.mp. 39579
6 adolescent*.mp. 477940
7 young adult*.mp. 207811
8 minor*.mp. 95495

9	boy*.mp.	82905
10	girl*.mp.	80139
11	exp Orphans/	1041
12	orphan*.mp.	3903
13	exp Adopted Children/	2026
14	adopted child*.mp.	2964
15	exp "Adoption (Child)"/	5681
16	adopt*.mp.	109707
17	fostered child*.mp.	71
18	teen*.mp.	25695
19	juvenile.mp.	39054
20	exp Sons/	1779
21	son*.mp.	44406
22	exp Daughters/	3587
23	daughter*.mp.	14280
24	youth.mp.	114071
25	school age.mp.	18544
26	young person.mp.	1573
27	young people.mp.	33689
28	exp Child Labor/	361
29	child labo?r.mp.	626
30	childhood.mp.	230615
31	or/2-30	1549716
32	1 and 31	167
33	exp Well Being/	55416
34	well-being.mp.	112373
35	psychological wellbeing.mp.	2008
36	exp Health/	394646
37	health.mp.	882062
38	exp Adolescent Health/	2984
39	adolescent health.mp.	8080
40	exp Child Health/	679
41	child* health.mp.	14025
42	infant health.mp.	1008
43	exp Infant Development/	23716
44	infant development.mp.	24770
45	exp Mental Health/	82517
46	mental health.mp.	264974
47	exp Oral Health/	1846
48	oral health.mp.	2083
49	exp Public Health/	33850
50	public health.mp.	67448
51	social determinants of health.mp.	2901
52	exp Physical Health/	7046
53	physical health.mp.	26261
54	exp Childhood Development/	118989
55	child* development.mp.	119910

56	exp Language Development/	31591
57	language development.mp.	38152
58	child* language.mp.	7493
59	mental capacity.mp.	1353
60	mental ability.mp.	2971
61	mental competency.mp.	2330
62	mental competency.mp.	2330
63	exp Motivation/	158497
64	motivation.mp.	158851
65	exp Safety/	33641
66	safety.mp.	83894
67	exp Psychological Development/	127157
68	psychological development.mp.	6547
69	exp Cognitive Development/	68261
70	cognitive development.mp.	36498
71	mental development.mp.	4249
72	exp Emotional Development/	8176
73	emotional development.mp.	11702
74	exp Psychosocial Development/	46483
75	psychosocial development.mp.	20763
76	social development.mp.	8246
77	educational development.mp.	1078
78	exp Social Behavior/	1250014
79	social behavio?r.mp.	90963
80	exp Aggressiveness/	4565
81	aggression.mp.	65811
82	exp Altruism/	4939
83	altruism.mp.	9001
84	competitive behavio?r.mp.	2439
85	exp Cooperation/	15992
86	cooperation.mp.	36521
87	cooperative behavio?r.mp.	10494
88	exp Deception/	12552
89	deception.mp.	11176
90	dehumanisation.mp.	94
91	exp Empowerment/	9069
92	empowerment.mp.	20641
93	exp Sexual Harassment/	2915
94	sexual harassment.mp.	4477
95	exp Help Seeking Behavior/	15519
96	help seeking behavio?r.mp.	7528
97	helping behavio?r.mp.	3428
98	incivility.mp.	1118
99	attitude*.mp.	524737
100	permissive*.mp.	4948
101	exp Prejudice/	9037
102	prejudice.mp.	22300

103	exp Self-Control/	12381
104	self-control.mp.	18744
105	non-sexual harassment.mp.	9
106	shy*.mp.	4653
107	social adaptation.mp.	1819
108	exp Social Adjustment/	9997
109	social adjustment.mp.	27220
110	social conformity.mp.	2466
111	exp Social Desirability/	3091
112	social desirability.mp.	12269
113	exp Social Discrimination/	15856
114	social discrimination.mp.	4402
115	exp Psychological Distance/	840
116	psychological distance.mp.	1467
117	exp Social Dominance/	1620
118	social dominance.mp.	4924
119	exp Social Isolation/	9113
120	social isolation.mp.	18072
121	exp Social Exclusion/	2059
122	social exclusion.mp.	4065
123	social marginalization.mp.	490
124	social competence.mp.	8259
125	exp Social Skills/	15423
126	social skill*.mp.	28673
127	social stigma.mp.	5704
128	exp Stigma/	16261
129	stigma.mp.	33054
130	stereotyp*.mp.	52998
131	educational status.mp.	16684
132	educational achievement.mp.	3217
133	exp Academic Achievement/	82433
134	academic achievement.mp.	83393
135	disabilit*.mp.	177248
136	mortality.mp.	49022
137	exp Morbidity/	7891
138	morbidity.mp.	28589
139	exp School Dropouts/	3254
140	school dropout*.mp.	3488
141	exp Dropouts/	6847
142	dropout*.mp.	15688
143	student dropout*.mp.	756
144	pregnant adolescent*.mp.	694
145	exp Adolescent Pregnancy/	3007
146	adolescent pregnancy.mp.	3375
147	teen* pregnancy.mp.	2048
148	teen* pregnancy.mp.	2048
149	crim*.mp.	106138

150	drug*.mp.	435466
151	exp Drug Abuse/	50358
152	drug abuse.mp.	57719
153	cigarette*.mp.	24188
154	smoking.mp.	65117
155	depressi*.mp.	399918
156	exp Suicide/	38893
157	suicid*.mp.	78433
158	exp Student Engagement/	8217
159	school engagement.mp.	1283
160	exp Intellectual Development/	4818
161	intellectual development.mp.	46233
162	exp Behavior Disorders/	62769
163	behavio?r disorder*.mp.	29477
164	character.mp.	50776
165	exp Personality/	504573
166	personality.mp.	334313
167	character disorder.mp.	358
168	exp Personality Disorders/	29164
169	personality disorder*.mp.	58586
170	identity.mp.	155237
171	personal life.mp.	2600
172	exp Self-Confidence/	4348
173	self-confidence.mp.	9820
174	confidence.mp.	91562
175	exp Empathy/	15747
176	empathy.mp.	34829
177	civic life.mp.	298
178	connect*.mp.	180331
179	competen*.mp.	126656
180	exp Nutrition/	67487
181	nutrition*.mp.	36763
182	exp Obesity/	27462
183	obes*.mp.	49605
184	vaccine*.mp.	6597
185	wellness.mp.	9455
186	longevity.mp.	6514
187	human flourishing.mp.	545
188	exp Happiness/	8971
189	happiness.mp.	21568
190	enjoyment.mp.	11989
191	exp Satisfaction/	67559
192	satisfaction.mp.	151949
193	personal satisfaction.mp.	9800
194	exp Job Satisfaction/	21261
195	job satisfaction.mp.	30733
196	exp Life Satisfaction/	13093
197	life satisfaction.mp.	20138

198 social well-being.mp. 1987
199 exp Spiritual Well Being/ 653
200 spiritual well-being.mp. 2200
201 physical well-being.mp. 2423
202 occupational well-being.mp. 200
203 economic well-being.mp. 810
204 financial well-being.mp. 514
205 environmental well-being.mp. 43
206 capability.mp. 14736
207 exp "Quality of Life"/ 49131
208 quality of life.mp. 100360
209 or/33-208 3610559
210 1 and 31 and 209 135

Social Policy and Practice <202304> (filter -ovid SP)

1 (Qualitative Comparative Analysis or Crisp set Qualitative Comparative Analysis or Fuzzy set Qualitative Comparative Analysis or QCA or csQCA or fsQCA or multi-value QCA).mp. [mp=abstract, title, publication type, heading word, accession number] 42
2 child*.mp. [mp=abstract, title, publication type, heading word, accession number] 117446
3 preschool child*.mp. [mp=abstract, title, publication type, heading word, accession number] 182
4 infant*.mp. [mp=abstract, title, publication type, heading word, accession number] 2529
5 newborn.mp. [mp=abstract, title, publication type, heading word, accession number] 187
6 adolescent*.mp. [mp=abstract, title, publication type, heading word, accession number] 11167
7 young adult*.mp. [mp=abstract, title, publication type, heading word, accession number] 3260
8 minor*.mp. [mp=abstract, title, publication type, heading word, accession number] 22492
9 boy*.mp. [mp=abstract, title, publication type, heading word, accession number] 3581
10 girl*.mp. [mp=abstract, title, publication type, heading word, accession number] 4385
11 orphan*.mp. [mp=abstract, title, publication type, heading word, accession number] 240
12 adopted child*.mp. [mp=abstract, title, publication type, heading word, accession number] 1539
13 fostered child*.mp. [mp=abstract, title, publication type, heading word, accession number] 185
14 teen*.mp. [mp=abstract, title, publication type, heading word, accession number] 4105
15 juvenile.mp. [mp=abstract, title, publication type, heading word, accession number] 1589
16 son*.mp. [mp=abstract, title, publication type, heading word, accession number] 820
17 daughter*.mp. [mp=abstract, title, publication type, heading word, accession number] 930
18 youth.mp. [mp=abstract, title, publication type, heading word, accession number] 16147
19 school age.mp. [mp=abstract, title, publication type, heading word, accession number] 588

20 young person.mp. [mp=abstract, title, publication type, heading word, accession number] 2091
21 young people.mp. [mp=abstract, title, publication type, heading word, accession number] 54339
22 child labo?r.mp. [mp=abstract, title, publication type, heading word, accession number] 328
23 childhood.mp. [mp=abstract, title, publication type, heading word, accession number] 8170
24 or/2-23 160957
25 1 and 24 18
26 wellbeing.mp. [mp=abstract, title, publication type, heading word, accession number] 11477
27 psychological wellbeing.mp. [mp=abstract, title, publication type, heading word, accession number] 235
28 health.mp. [mp=abstract, title, publication type, heading word, accession number] 132543
29 adolescent health.mp. [mp=abstract, title, publication type, heading word, accession number] 176
30 child* health.mp. [mp=abstract, title, publication type, heading word, accession number] 1593
31 infant health.mp. [mp=abstract, title, publication type, heading word, accession number] 48
32 mental health.mp. [mp=abstract, title, publication type, heading word, accession number] 45151
33 oral health.mp. [mp=abstract, title, publication type, heading word, accession number] 150
34 public health.mp. [mp=abstract, title, publication type, heading word, accession number] 10266
35 physical health.mp. [mp=abstract, title, publication type, heading word, accession number] 2614
36 language development.mp. [mp=abstract, title, publication type, heading word, accession number] 135
37 child* development.mp. [mp=abstract, title, publication type, heading word, accession number] 3924
38 child* language.mp. [mp=abstract, title, publication type, heading word, accession number] 57
39 mental capacity.mp. [mp=abstract, title, publication type, heading word, accession number] 1531
40 mental competency.mp. [mp=abstract, title, publication type, heading word, accession number] 1
41 motivation.mp. [mp=abstract, title, publication type, heading word, accession number] 3049
42 safety.mp. [mp=abstract, title, publication type, heading word, accession number] 15583
43 psychological development.mp. [mp=abstract, title, publication type, heading word, accession number] 72
44 mental development.mp. [mp=abstract, title, publication type, heading word, accession number] 19
45 emotional development.mp. [mp=abstract, title, publication type, heading word, accession number] 298
46 social development.mp. [mp=abstract, title, publication type, heading word, accession number] 737

47 educational development.mp. [mp=abstract, title, publication type, heading word, accession number] 84
48 social development.mp. [mp=abstract, title, publication type, heading word, accession number] 737
49 educational development.mp. [mp=abstract, title, publication type, heading word, accession number] 84
50 social behavio?r.mp. [mp=abstract, title, publication type, heading word, accession number] 4412
51 aggression.mp. [mp=abstract, title, publication type, heading word, accession number] 2460
52 altruism.mp. [mp=abstract, title, publication type, heading word, accession number] 128
53 competitive behavio?r.mp. [mp=abstract, title, publication type, heading word, accession number] 16
54 cooperation.mp. [mp=abstract, title, publication type, heading word, accession number] 6129
55 cooperative behavio?r.mp. [mp=abstract, title, publication type, heading word, accession number] 4
56 decepti*.mp. [mp=abstract, title, publication type, heading word, accession number] 125
57 dehumanization.mp. [mp=abstract, title, publication type, heading word, accession number] 7
58 empowerment.mp. [mp=abstract, title, publication type, heading word, accession number] 6214
59 sexual harassment.mp. [mp=abstract, title, publication type, heading word, accession number] 589
60 help seeking behavio?r.mp. [mp=abstract, title, publication type, heading word, accession number] 265
61 helping behavio?r.mp. [mp=abstract, title, publication type, heading word, accession number] 43
62 incivil*.mp. [mp=abstract, title, publication type, heading word, accession number] 35
63 attitude.mp. [mp=abstract, title, publication type, heading word, accession number] 5506
64 prejudice.mp. [mp=abstract, title, publication type, heading word, accession number] 915
65 self-control.mp. [mp=abstract, title, publication type, heading word, accession number] 166
66 shy*.mp. [mp=abstract, title, publication type, heading word, accession number]82
67 social adaptation.mp. [mp=abstract, title, publication type, heading word, accession number] 22
68 social adjustment.mp. [mp=abstract, title, publication type, heading word, accession number] 83
69 social conformity.mp. [mp=abstract, title, publication type, heading word, accession number] 7
70 social desirability.mp. [mp=abstract, title, publication type, heading word, accession number] 66
71 social discrimination.mp. [mp=abstract, title, publication type, heading word, accession number] 13
72 psychological distance.mp. [mp=abstract, title, publication type, heading word, accession number] 5
73 social dominance.mp. [mp=abstract, title, publication type, heading word, accession number] 13
74 social isolation.mp. [mp=abstract, title, publication type, heading word, accession number] 2465

75 social exclusion.mp. [mp=abstract, title, publication type, heading word, accession number] 15723
76 social marginalization.mp. [mp=abstract, title, publication type, heading word, accession number] 12
77 social competence.mp. [mp=abstract, title, publication type, heading word, accession number] 151
78 social skills.mp. [mp=abstract, title, publication type, heading word, accession number] 1272
79 social stigma.mp. [mp=abstract, title, publication type, heading word, accession number] 105
80 stereotyp*.mp. [mp=abstract, title, publication type, heading word, accession number] 3515
81 educational status.mp. [mp=abstract, title, publication type, heading word, accession number] 150
82 cognitive development.mp. [mp=abstract, title, publication type, heading word, accession number] 235
83 disabil*.mp. [mp=abstract, title, publication type, heading word, accession number] 35727
84 mortality.mp. [mp=abstract, title, publication type, heading word, accession number] 3937
85 morbidity.mp. [mp=abstract, title, publication type, heading word, accession number] 1400
86 school dropout*.mp. [mp=abstract, title, publication type, heading word, accession number] 67
87 dropout*.mp. [mp=abstract, title, publication type, heading word, accession number] 241
88 adolescent pregnancy.mp. [mp=abstract, title, publication type, heading word, accession number] 40
89 crim*.mp. [mp=abstract, title, publication type, heading word, accession number] 22682
90 drug*.mp. [mp=abstract, title, publication type, heading word, accession number] 12185
91 cigarette*.mp. [mp=abstract, title, publication type, heading word, accession number] 352
92 depressi*.mp. [mp=abstract, title, publication type, heading word, accession number] 11424
93 suicid*.mp. [mp=abstract, title, publication type, heading word, accession number] 3833
94 suicid* attempt.mp. [mp=abstract, title, publication type, heading word, accession number] 124
95 school engagement.mp. [mp=abstract, title, publication type, heading word, accession number] 54
96 academic achievement.mp. [mp=abstract, title, publication type, heading word, accession number] 359
97 intellectual development.mp. [mp=abstract, title, publication type, heading word, accession number] 21
98 behavio?r disorder.mp. [mp=abstract, title, publication type, heading word, accession number] 17
99 behavio?r disorder.mp. [mp=abstract, title, publication type, heading word, accession number] 17
100 character.mp. [mp=abstract, title, publication type, heading word, accession number] 1071

101 identity.mp. [mp=abstract, title, publication type, heading word, accession number] 8091
102 personal life.mp. [mp=abstract, title, publication type, heading word, accession number] 125
103 confidence.mp. [mp=abstract, title, publication type, heading word, accession number] 5490
104 empathy.mp. [mp=abstract, title, publication type, heading word, accession number] 1396
105 civic life.mp. [mp=abstract, title, publication type, heading word, accession number] 48
106 connect*.mp. [mp=abstract, title, publication type, heading word, accession number] 8073
107 competen*.mp. [mp=abstract, title, publication type, heading word, accession number] 5354
108 nutrition*.mp. [mp=abstract, title, publication type, heading word, accession number] 2634
109 obes*.mp. [mp=abstract, title, publication type, heading word, accession number] 2227
110 vaccine*.mp. [mp=abstract, title, publication type, heading word, accession number] 249
111 teen* pregnancy.mp. [mp=abstract, title, publication type, heading word, accession number] 1095
112 wellness.mp. [mp=abstract, title, publication type, heading word, accession number] 328
113 longevity.mp. [mp=abstract, title, publication type, heading word, accession number] 817
114 human flourishing.mp. [mp=abstract, title, publication type, heading word, accession number] 12
115 happiness.mp. [mp=abstract, title, publication type, heading word, accession number] 890
116 satisfaction.mp. [mp=abstract, title, publication type, heading word, accession number] 7124
117 personal satisfaction.mp. [mp=abstract, title, publication type, heading word, accession number] 23
118 life satisfaction.mp. [mp=abstract, title, publication type, heading word, accession number] 1349
119 social well-being.mp. [mp=abstract, title, publication type, heading word, accession number] 288
120 spiritual well-being.mp. [mp=abstract, title, publication type, heading word, accession number] 64
121 physical well-being.mp. [mp=abstract, title, publication type, heading word, accession number] 144
122 intellectual well-being.mp. [mp=abstract, title, publication type, heading word, accession number] 4
123 occupational well-being.mp. [mp=abstract, title, publication type, heading word, accession number] 2
124 economic well-being.mp. [mp=abstract, title, publication type, heading word, accession number] 298
125 financial well-being.mp. [mp=abstract, title, publication type, heading word, accession number] 70
126 environmental well-being.mp. [mp=abstract, title, publication type, heading word, accession number] 38
127 capab*.mp. [mp=abstract, title, publication type, heading word, accession number] 3021
128 capability.mp. [mp=abstract, title, publication type, heading word, accession number] 1347

129 quality of life.mp. [mp=abstract, title, publication type, heading word, accession number] 13105
130 social determinants of health.mp. [mp=abstract, title, publication type, heading word, accession number] 257
131 or/26-130 241402
132 1 and 24 and 131 9

3.6 SCOPUS

Database name	Scopus
Database platform	Clarivate
Data searched	1 September 2022
Searched by	ATK
Number of hits	67

#1	"Qualitative Comparative Analysis" OR "Crisp set Qualitative Comparative Analysis" OR "Fuzzy set Qualitative Comparative Analysis" OR "multi-value QCA"	3,494	4425
#2	child* OR adolescent* OR "young adult or minor*" OR boy* OR girl* OR orphan* OR juvenile OR son* OR daughter* OR youth OR "young people" OR "child labo?r" OR childhood	6,073,096	6,291,383
#3	wellbeing OR "psychological wellbeing" OR health OR "adolescent health" OR "infant health" OR "mental health" OR "public health" OR "physical well-being" OR "child development" OR "child* language" OR "mental competency" OR "psychological development" OR "social development" OR "educational development" OR "educational achievement" OR disability OR "school dropout" OR "adolescent pregnancy" OR crime OR depression OR suicide OR "academic achievement" OR "intellectual achievement" OR “confidence” OR “empathy” OR “nutrition* “ OR obes* OR vaccine* OR wellness OR "human flourishing" OR happiness OR "life satisfaction" OR "social wellbeing" OR "spiritual wellbeing" OR "physical wellbeing" OR "intellectual wellbeing" OR "occupational wellbeing" OR "economic wellbeing" OR "financial wellbeing" OR "environmental wellbeing" OR capab* OR "quality of life"	10,684,347	11,338,251
#5	("Qualitative Comparative Analysis" OR "Crisp set Qualitative Comparative Analysis" OR "Fuzzy set Qualitative Comparative Analysis" OR "multi-value QCA") AND (child* OR adolescent* OR "young adult or minor*" OR boy* OR girl* OR orphan* OR juvenile OR son* OR daughter* OR youth OR "young people" OR "child labo?r" OR childhood)) AND (wellbeing OR "psychological wellbeing" OR health OR "adolescent health" OR "infant	67	72

	health" OR "mental health" OR "public health" OR "physical well-being" OR "child development" OR "child* language" OR "mental competency" OR "psychological development" OR "social development" OR "educational development" OR "educational achievement" OR disability OR "school dropout" OR "adolescent pregnancy" OR crime OR depression OR suicide OR "academi c achievement" OR "intellectual achievement" OR "confidence" OR "empathy" OR "nutrition* " OR obes* OR vaccine* OR wellness OR "human flourishing" OR happiness OR "life satisfaction" OR "social wellbeing" OR "spiritual wellbeing" OR "physical wellbeing" OR "intellectual wellbeing" OR "occupational wellbeing" OR "economic wellbeing" OR "financial wellbeing" OR "environmental wellbeing" OR capab* OR "quality of life")		

3.7 Web of Science

Database name	Web of Science
Database platform	Clarivate Analytics
Dates of database coverage	1970-2023
Data searched	1 September 2022, 2 june 2023
Searched by	ATK
Number of hits	101

**#1**	"Qualitative Comparative Analysis" OR "Crisp set Qualitative Comparative Analysis" OR "Fuzzy set Qualitative Comparative Analysis" OR "multi-value QCA"	3077<br>3,611
**#2**	child* OR adolescent* OR "young adult or minor*" OR boy* OR girl* OR orphan* OR juvenile OR son* OR daughter* OR youth OR "young people" OR "child labo?r" OR childhood	**5,773,537**<br>**5,989,433**
**#3**	wellbeing OR "psychological wellbeing" OR health OR "adolescent health" OR "infant health" OR "mental health" OR "public health" OR "physical well-being" OR "child development" OR "child* language" OR "mental competency" OR "psychological development" OR "social development" OR "educational development" OR "educational achievement" OR disability OR "school dropout" OR "adolescent pregnancy" OR crime OR depression OR suicide OR "academic achievement" OR "intellectual achievement" OR “confidence” OR “empathy” OR “nutrition* “ OR obes* OR vaccine* OR wellness OR "human flourishing" OR happiness OR "life satisfaction" OR "social wellbeing" OR "spiritual wellbeing" OR "physical wellbeing" OR "intellectual wellbeing" OR "occupational wellbeing" OR "economic	**12,680,836**<br><br>13,206,443

	wellbeing" OR "financial wellbeing" OR "environmental wellbeing" OR capab* OR "quality of life"	
#5	("Qualitative Comparative Analysis" OR "Crisp set Qualitative Comparative Analysis" OR "Fuzzy set Qualitative Comparative Analysis" OR "multi-value QCA") AND (child* OR adolescent* OR "young adult or minor*" OR boy* OR girl* OR orphan* OR juvenile OR son* OR daughter * OR youth OR "young people" OR "child labo?r" OR childhood)) AND (wellbeing OR "psychological wellbeing" OR health OR "adolescent health" OR "infant health" OR "mental health" OR "public health" OR "physical well-being" OR "child development" OR "child* language" OR "mental competency" OR "psychological development" OR "social development" OR "educational development" OR "educational achievement" OR disability OR "school dropout" OR "adolescent pregnancy" OR crime OR depression OR suicide OR "academic achievement" OR "intellectual achievement" OR "confidence" OR "empathy" OR "nutrition* " OR obes* OR vaccine* OR wellness OR "human flourishing" OR happiness OR "life satisfaction" OR "social wellbeing" OR "spiritual wellbeing" OR "physical wellbeing" OR "intellectual wellbeing" OR "occupational wellbeing" OR "economic wellbeing" OR "financial wellbeing" OR "environmental wellbeing" OR capab* OR "quality of life")	123
