# Supplementary material for: The use of Qualitative Comparative Analysis (QCA) in child well-being research: a scoping review of research on child well-being research and interventions
Source: BMC Public Health. 2025 Sep 25;25:3122. doi: 10.1186/s12889-025-23821-x (PMC12462042; doi:10.1186/s12889-025-23821-x)
Supplement: Supplementary file 4 — Supplementary Material 4. [file 12889_2025_23821_MOESM4_ESM.pdf]

Excluded systematic reviews/meta-analyses employing QCA.

1	Forman-Hoffman VL, Middleton JC, McKeeman JL, Stambaugh LF, Christian RB, Gaynes BN, Kane HL, Kahwati LC, Lohr KN, Viswanathan M. Quality improvement, implementation, and dissemination strategies to improve mental health care for children and adolescents: a systematic review. Implement Sci. 2017 Jul 24;12(1):93. doi: 10.1186/s13012-017-0626-4. PMID: 28738821; PMCID: PMC5525230.
2	Melendez-Torres GJ, Sutcliffe K, Burchett HED, Rees R, Richardson M, Thomas J. Weight management programmes: Re-analysis of a systematic review to identify pathways to effectiveness. Health Expect. 2018 Jun;21(3):574-584. doi: 10.1111/hex.12667. Epub 2018 Mar 5. PMID: 29508524; PMCID: PMC5980502.
3	Burchett HED, Sutcliffe K, Melendez-Torres GJ, Rees R, Thomas J. Lifestyle weight management programmes for children: A systematic review using Qualitative Comparative Analysis to identify critical pathways to effectiveness. Prev Med. 2018 Jan;106:1-12. https://doi: 10.1016/j.ypmed.2017.08.025 Epub 2017 Sep 1. PMID: 28865809.
4	Thongseiratch T, Leijten P, Melendez-Torres GJ. Online parent programs for children's behavioral problems: a meta-analytic review. Eur Child Adolesc Psychiatry. 2020;29(11):1555-1568. https://doi:10.1007/s00787-020-01472-0
5	Moore, D.A., Russell, A.E., Matthews, J., Ford, T.J., Rogers, M., Ukoumunne, O.C., Kneale, D., Thompson-Coon, J., Sutcliffe, K., Nunns, M., Shaw, L. and Gwernan-Jones, R. (2018), School-based interventions for attention-deficit/hyperactivity disorder: A systematic review with multiple synthesis methods. Rev Educ, 6: 209-263. https://doi.org/10.1002/rev3.3149
6	Hanckel, B., Petticrew, M., Thomas, J. et al. The use of Qualitative Comparative Analysis (QCA) to address causality in complex systems: a systematic review of research on public health interventions. BMC Public Health 21, 877 (2021). https://doi.org/10.1186/s12889-021-10926-2
7	Thongseiratch T, Chalermphol K, Traipidok P, Charleowsak P. Promoting medication adherence in children with attention-deficit/hyperactivity disorder: a mixed-methods systematic review with meta-analysis and qualitative comparative analysis. J Atten Disord. 2024;28(2):139-150. doi:10.1177/10870547231211021
8	Harris K, Kneale D, Lasserson TJ, McDonald VM, Grigg J, Thomas J. School-based self-management interventions for asthma in children and adolescents: a mixed methods systematic review. Cochrane Database Syst Rev. 2019 Jan 28;1(1):CD011651. doi: 10.1002/14651858.CD011651.pub2. PMID: 30687940; PMCID: PMC6353176.
